# Increasing Acyl CoA thioesterase activity alters phospholipid profile without effect on insulin action in skeletal muscle of rats

**DOI:** 10.1038/s41598-018-32354-w

**Published:** 2018-09-18

**Authors:** Ishita Bakshi, Simon H. J. Brown, Amanda E. Brandon, Eurwin Suryana, Todd W. Mitchell, Nigel Turner, Gregory J. Cooney

**Affiliations:** 1 Diabetes and Metabolism Division, Garvan Institute, Sydney, Australia; 2 School of Biological Sciences, University of Wollongong, Wollongong, Australia; 3 Sydney Medical School, Charles Perkins Centre, The University of Sydney, Sydney, Australia; 4 Department of Pharmacology, School of Medical Sciences, UNSW Sydney, Sydney, Australia

## Abstract

Increased lipid metabolism in muscle is associated with insulin resistance and therefore, many strategies have been employed to alter fatty acid metabolism and study the impact on insulin action. Metabolism of fatty acid requires activation to fatty acyl CoA by Acyl CoA synthases (ACSL) and fatty acyl CoA can be hydrolysed by Acyl CoA thioesterases (Acot). Thioesterase activity is low in muscle, so we overexpressed Acot7 in muscle of chow and high-fat diet (HFD) rats and investigated effects on insulin action. Acot7 overexpression modified specific phosphatidylcholine and phosphatidylethanolamine species in tibialis muscle of chow rats to levels similar to those observed in control HFD muscle. The changes in phospholipid species did not alter glucose uptake in tibialis muscle under hyperinsulinaemic/euglycaemic clamped conditions. Acot7 overexpression in white extensor digitorum longus (EDL) muscle increased complete fatty acid oxidation *ex-vivo* but was not associated with any changes in glucose uptake *in-vivo*, however overexpression of Acot7 in red EDL reduced insulin-stimulated glucose uptake *in-vivo* which correlated with increased incomplete fatty acid oxidation *ex-vivo*. In summary, although overexpression of Acot7 in muscle altered some aspects of lipid profile and metabolism in muscle, this had no major effect on insulin-stimulated glucose uptake.

## Introduction

Insulin resistance and lipid accumulation in skeletal muscle are major risk factors for the development of Type 2 Diabetes^1^. In rodents fed a high fat diet (HFD), lipid accumulation in muscle requires circulating fatty acids to be transported into the muscle through fatty acyl transfer proteins (FATP) or CD36^2^. After transport, fatty acids require activation to their acyl CoA forms before being metabolised by either anabolic processes like synthesis of triglycerides and other complex lipids, or catabolic processes like β-oxidation. Activation can be carried out by FATP (also known as ACSVL) proteins or by different isoforms of Acyl CoA synthase (ACSL) localised to cellular membranes^3,4^. Cells also have a class of enzymes called Acyl CoA thioesterases (Acot) that can remove the CoA group from fatty acyl CoA. It has been suggested that the opposing activity of ACSL and Acots may regulate CoA and fatty acid levels in cells, or even channel fatty acids into specific pathways depending on fatty acid chain length, specificity and subcellular localisation^3,5^. Channelling fatty acids into synthesis of complex lipids may have downstream effects on metabolism and insulin signalling^6,7^.

The Acot enzymes have only recently been classified and characterised^8–10^ and their functional relevance is still being elucidated. Through either gain or loss of function studies, different Acots have been suggested to play important roles in triglyceride storage^11,12^, fatty acid oxidation^11–16^, mitochondrial function^15–17^, energy expenditure^12,14^, insulin signalling^17,18^ and even lipid synthesis^14^ in brown adipose, liver and heart tissues. Changes in these pathways are also reported to occur in skeletal muscle of HFD fed animals and have been speculated to either contribute to insulin resistance^19,20^, or alleviate it^21–24^. The aim of this study was to use overexpression of cytoplasmic Acot7a^25,26^ to increase total muscle thioesterase activity and examine the effects on lipid composition and insulin-stimulated glucose uptake in skeletal muscle from chow and HFD fed animals.

## Results

### Acute overexpression of Acot7

**Figure 1:**
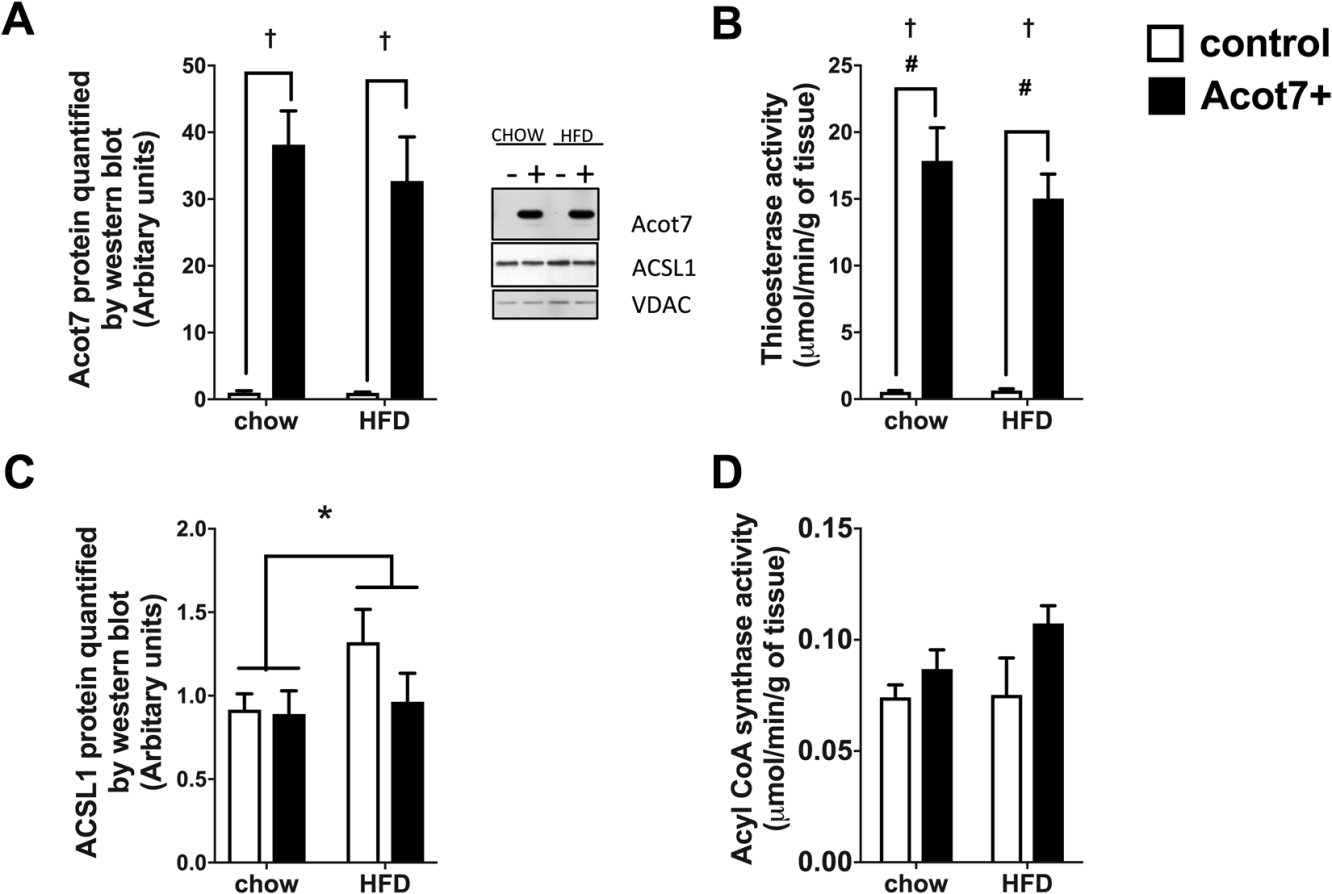
Overexpression of AAV9-tMCK-Acot7 and its effect on the opposing ACSL enzyme in tibialis muscle. Acot7 protein levels (**A**), thioesterase activity (**B**) ACSL1 protein level (**C**) Acyl-CoA synthase activity (**D**). Data is mean ± SEM, *p < 0.05, 2-way ANOVA, diet; ^†^p < 0.01 2-way ANOVA, Acot7; ^#^p < 0.01 post-hoc Holm and Sidak test, (n = 6).

Acot7 protein is expressed at relatively low levels in different skeletal muscles under chow fed conditions (Supplementary Fig. S1) and total thioesterase activity is also low (Fig. 1B). Therefore, to increase thioesterase activity in muscle, an N-terminal Flag-tagged Acot7 construct, AAV9-tMCK-Acot7, was injected into the right tibialis muscle of rats, which were then fed chow or high fat diet (HFD) for 4 weeks. Preliminary studies using AAV-GFP determined that after 4 weeks, 70–80% of the muscle fibres were transduced (data not shown) and 4 weeks after injection with AAV9-tMCK-Acot7, ACOT7 protein was significantly increased in the right tibialis muscle in both chow and HFD fed rats (Fig. 1A). This overexpression of Acot7 increased total thioesterase activity from 0.5 to 16µmol/min/g tissue on average (Fig. 1B). Overexpression of Acot7 might be expected to cleave the CoA group from fatty acids and alter the lipid profile in the muscle, an effect that could be limited if there was a compensatory increase in the opposing enzyme ACSL. Therefore, the effect of Acot7 overexpression on the predominant ACSL enzyme, ACSL1 protein and total ACSL activity was determined in chow and HFD animals. HFD increased ACSL1 protein levels, which did not translate into an increased ACSL activity. There was no effect of Acot7 overexpression on the ACSL1 protein levels (Fig. 1C) or overall ACSL enzyme activity (p = 0.08) (Fig. 1D). Therefore, any impact of Acot7 overexpression is unlikely to be limited by an increase in ACSL enzymes.

### Effect of Acot7 overexpression on lipid profile

Long chain acyl CoA (LCAC) levels have been associated with impairment in insulin action in muscle^27^ and Acot7 is known to have a substrate specificity for LCAC^26^. To determine if overexpressing Acot7 had downstream effects on lipid profile, total LCAC levels were measured in the red quad muscle and a comprehensive lipidomic analysis was performed in tibialis muscle from chow and HFD rats overexpressing Acot7 and compared to the contralateral control muscle. LCAC levels increased with HFD but were not altered by Acot7 overexpression (Supplementary Fig. S2).

Overall the lipidomic analysis of tibialis muscle identified 235 long chain (C16–22) lipid species. The total amount of major lipid classes detected in tibialis muscle from the two diets and Acot7 treatment is displayed in Table 1. Total levels of triglycerides (TAG) and phosphatidylglycerol (PG) was increased with HFD, whereas free cholesterol (FC), phosphatidylserine (PS), phosphatidylethanolamine (PE), phosphatidylcholine (PC) and sphingomyelin (SM) were decreased with HFD. Acot7 overexpression reduced total levels of cardiolipin, PE and PS species.

**Table 1:** Alteration in total lipids within each class in tibialis muscle with diet or Acot7.

Total lipids	Control Chow	Acot7 Chow	Control HFD	Acot7 HFD	2 Way ANOVA		
	(nmol/g)	(nmol/g)	(nmol/g)	(nmol/g)	Interaction	Diet	Acot7
CE	28.9 ± 2.9	73.5 ± 33.6	55.3 ± 9	44.1 ± 3.6			
Cer	47.6 ± 4.1	45.2 ± 1.4	44.9 ± 1.3	44 ± 2			
CL	1853.3 ± 104.5	1308.7 ± 72.3^#^	1571.7 ± 164	1419.6 ± 96			*
DAG	1103.3 ± 68.3	1044.5 ± 54.9	1210.8 ± 57.8	1155.8 ± 101.9			
FC	2282.5 ± 179.9	1883.1 ± 135.5	1972.5 ± 114.8	1629.8 ± 109.9		*	
LPC	192.5 ± 7.7	159 ± 8	196.6 ± 11.4	186.2 ± 11.2			
PC	15958.5 ± 927.9	12672.9 ± 600.9	14151.1 ± 491.8	13941.2 ± 762.6	*	*	
PE	5971.8 ± 245.2	4421 ± 196.8^##^	4535.4 ± 234.1	4676 ± 232.5	**	**	*
PG	103 ± 3.9	88.5 ± 5.8	123.5 ± 5.8	123.7 ± 8.8		**	
PS	435.4 ± 16	332.7 ± 15.4^##^	337.6 ± 13.7	334.3 ± 18.4	*	**	*
DSHM	6.8 ± 2.2	9.8 ± 1.8	4.2 ± 1.2	6.3 ± 1.6			
SM	957.4 ± 73	813.9 ± 77.5	708.5 ± 63.4	790.5 ± 42.4		*	
TAG	2843.1 ± 322.4	4407.9 ± 628.6	5847 ± 1027.6	6245.1 ± 442.5		**	

Total lipid content in each group. Data is mean ± SEM, *p < 0.05, **p < 0.01, 2-way ANOVA ^#^p < 0.05, ^##^p < 0.01 post hoc Sidak test.

**Figure 2:**
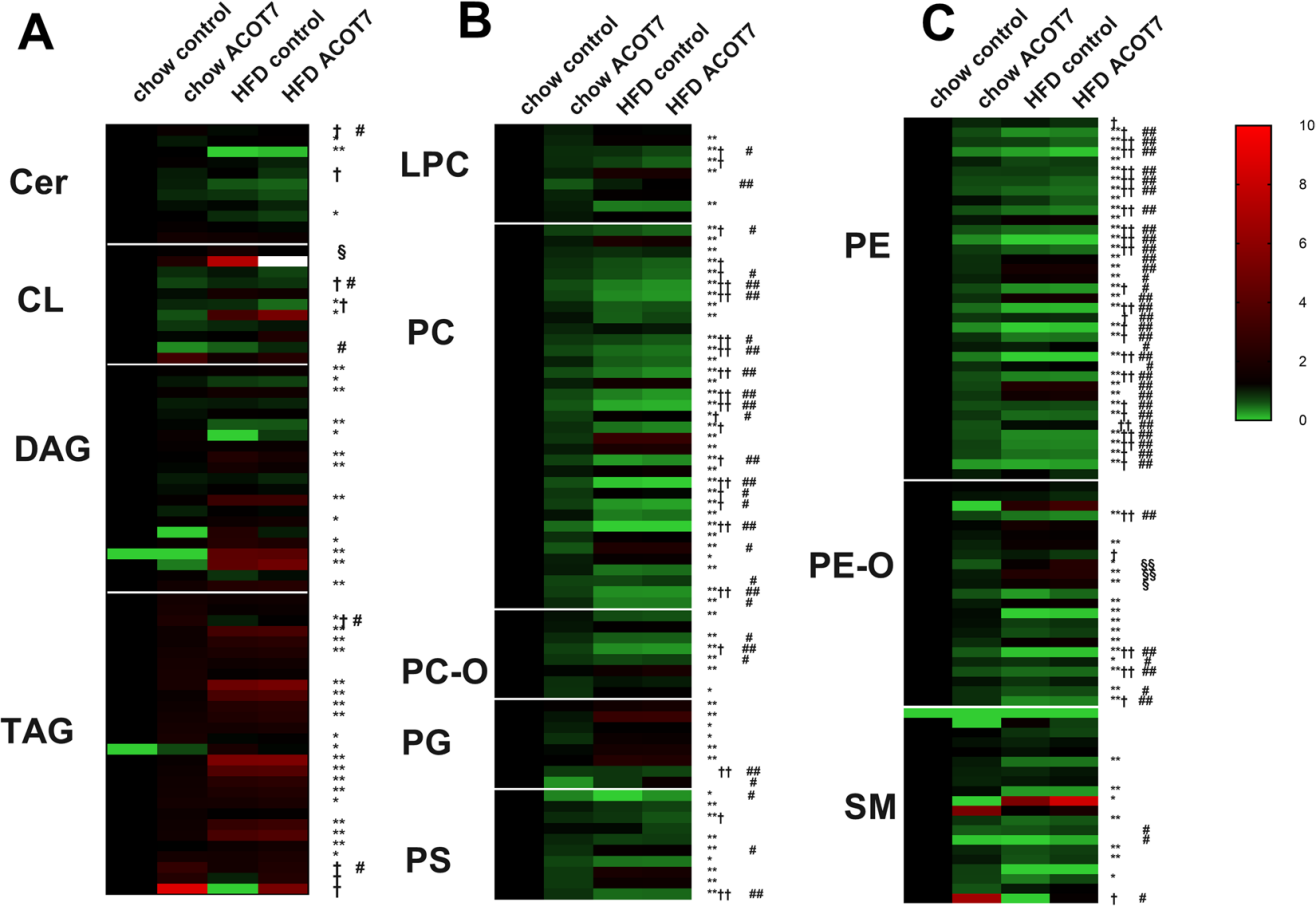
Effect of AAV9-tMCK-Acot7 overexpression on lipid profile in tibialis muscle. Ceramide (Cer), cardiolipin (CL), diacylglyceride (DAG), triacylglyceride (TAG) (**A**); lysophosphatidylcholine (LPC), phosphatidylcholine (PC), ether-linked phosphatidylcholine (PC-O), phosphatidylglycerol (PG), phosphatidylserine (PS), (**B**) phosphatidylethanolamine (PE), ether-linked phosphatidylethanolamine (PE-O), sphingomyelin (SM) (**C**). Data is fold difference over control chow; *p < 0.05, 2-way ANOVA, diet; ^†^p < 0.05, 2-way ANOVA, Acot7; ^#^p < 0.05, post-hoc Holm and Sidak test for chow, ^§^p < 0.05, post-hoc Holm and Sidak test for HFD animals (n = 6).

A heatmap of the lipid profile of the four groups of muscle, control chow, Acot7 overexpressing chow, control HFD and Acot7 overexpressing HFD was created to obtain an overview of the effects of diet and Acot7 on complex lipids in the tibialis muscle. HFD altered 150 species, while Acot7 overexpression altered 88 species out of 235 species detected (Fig. 2), as estimated by 2-way ANOVA (63 species) or post hoc test (79 species). Acot7 overexpression had opposing effects in chow compared to HFD muscle for some of the lipid species. For these species, the effects of Acot7 was not detected by 2-way ANOVA analysis, but were significantly different when compared using a post-hoc test.

Acot7 overexpression altered 75 lipid species in the chow fed animals and only 4 species in the HFD animals. The major effect of increasing Acot7 activity on phospholipid profile appeared to be similar to the effect of the HFD with the same lower levels of certain phospholipid species compared to control chow muscle. This was predominately observed in PC and PE lipid classes (50 out of the 59 PC and PE species altered by Acot7 overexpression, were reduced towards, or to levels observed in HFD control muscle) (Fig. 2). Conversely, levels of certain triglyceride species were higher with Acot7 overexpression in chow and HFD fed animals, but the increase due to Acot7 only reached statistical significance for TAG 56:8, TAG 58:8, TAG 58:9.

The lipid classes (PC, PE, PS) that were altered by Acot7 overexpression in the 2-way ANOVA analysis, were further analysed on the basis of the percentage of saturated fatty acid (SFA), mono-unsaturated fatty acid (MUFA) or poly-unsaturated fatty acid (PUFA) contributing to total fatty acid content of each lipid class to determine if the HFD and Acot7 overexpression were changing the composition as well as the amount of different phospholipids. For a more specific analysis, PC and PE species were divided into PC, ether-linked PC (PC-O), PE and ether linked-PE (PE-O) respectively. Since the individual fatty acids in the cardiolipin species could not be determined, this lipid species could not be grouped in this way and was not further analysed.

**Figure 3:**
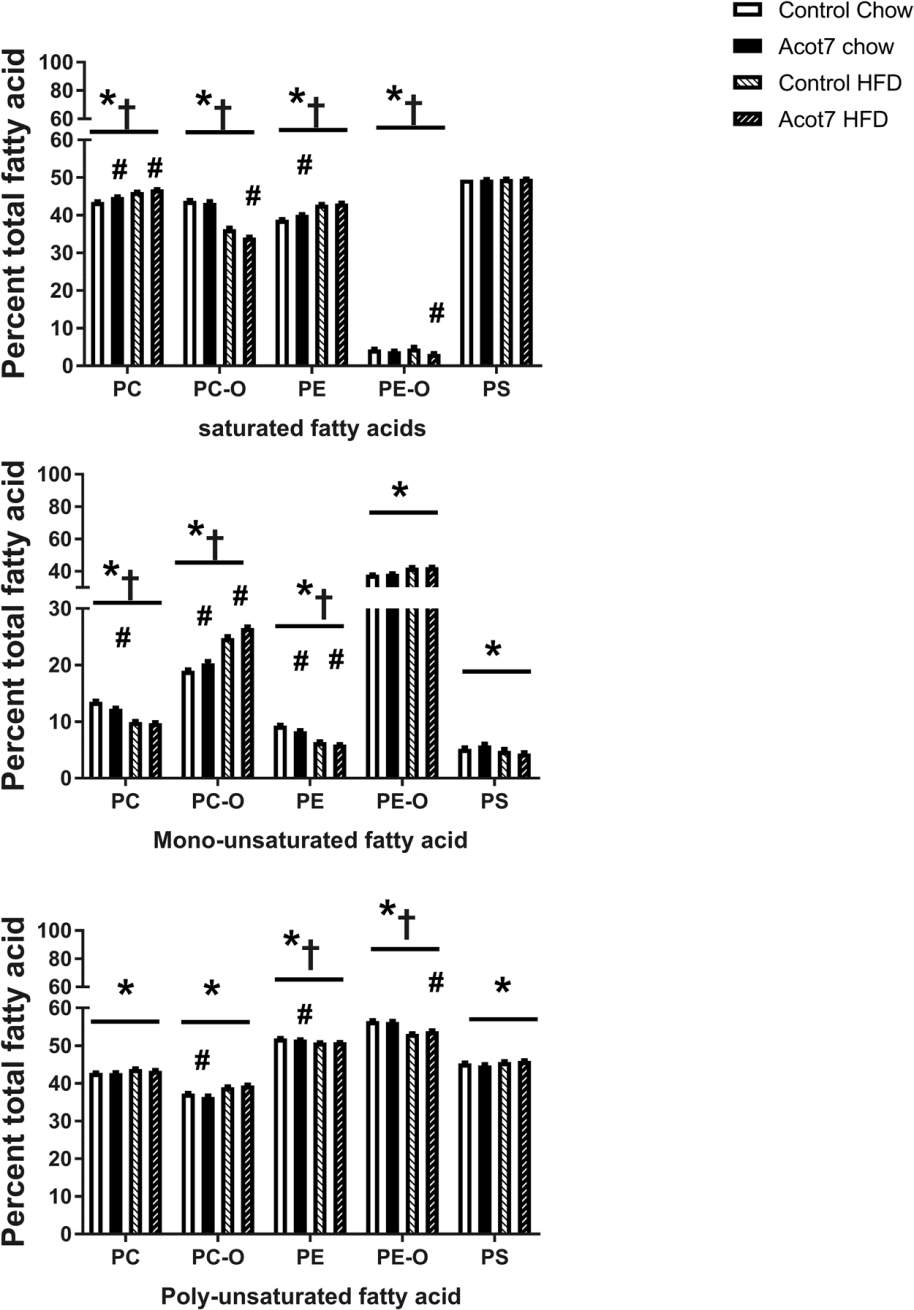
Effect of AAV9-tMCK-Acot7 overexpression on saturated, MUFA and PUFA content of phospholipids in tibialis muscle. Fatty acid composition of each lipid class as a percent of total fatty acid content of PC (phosphatidylcholine), PC-O (ether-linked phosphatidylcholine), PE (phosphatidylethanolamine), PE-O (ether-linked phorsphatidylethanolamine), PS (phosphatidylserine) lipid classes. Content of saturated fatty acid (SFA) (**A**) monounsaturated fatty acid (MUFA) (**B**) polyunsaturated fatty acid (PUFA) (**C**) are presented as the mean ± SEM for n = 6. *p < 0.05, 2-way ANOVA for effect of diet; ^†^p < 0.05, 2-way ANOVA for effect of Acot7, ^#^p < 0.05, post-hoc Holm and Sidak test for the effect of Acot7 overexpression within the Control Chow or HFD fed rats.

The HFD significantly increased the percentage of SFA in PC and PE but not PS while the percentage of SFA in PC-O and PE-O decreased (Fig. 3A). Acot7 overexpression also influenced the SFA composition of all species examined across chow and HFD except PS but post-hoc test determined that, apart from PC, the Acot7 effect were due to changes mainly in muscle from the chow control diet. A similar result was observed for the percent MUFA and PUFA in the various phospholipids examined with the HFD altering composition in all phospholipids while the effect of Acot7 overexpression was more selective and in most cases due to a greater effect in animals on a chow diet (Fig. 3B,C).

Further comparisons of changes in individual lipid species from other lipid classes highlighted that Acot7 and HFD treatments together altered only three lipid species (LPC 18:1, LPC 18:2, TAG 48:3), while 14 species were altered by Acot7 overexpression alone (Supplementary Table S1). Similar to the effects observed in PC and PE classes of lipids, most of the other lipid species altered by Acot7 overexpression were reduced only in chow fed animals (Fig. 2, Supplementary Table S1). Conversely, TAG 48:3, TAG 56:8, TAG 58:8, TAG 58:9, Cer 16:0, SM 26:2 were increased with Acot7 overexpression in chow fed tibialis. CL 72:5 is one of only 4 lipid species that was altered in the Acot7 overexpressing HFD tibialis. This was the only lipid species in our lipidomic analysis of the HFD muscle that was restored to the level of the control chow muscle, by Acot7 overexpression, without Acot7 having any effect on the CL 72:5 levels in the tibialis muscle from chow fed rats (Supplementary Table S1).

The effect of Acot7 overexpression in muscle of chow fed animals can be summarised as reducing the level of most species towards, or to the level observed in muscle from HFD rats. There was little or no additional effect of overexpression of Acot7 on lipid species in muscle from HFD rats, with only a small effect of Acot7 overexpression to increase levels of three PE-O species and CL 72:5 species towards the level observed in Acot7 overexpressing muscle from chow rats.

### Effect of Acot7 overexpression on muscle metabolism

**Figure 4:**
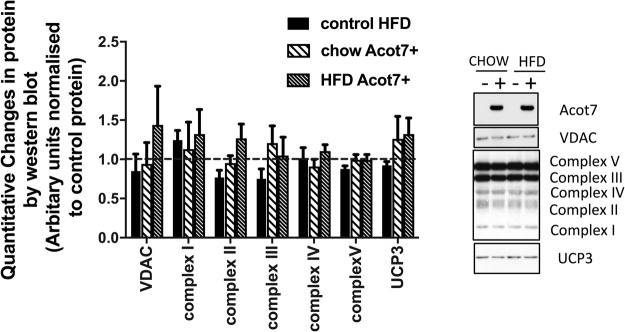
Effect of AAV9-tMCK-Acot7 on mitochondrial proteins in tibialis muscle. The dashed line indicates the level of the protein for control chow group. The protein levels of all other groups (control HFD, Acot7 overexpressing chow, Acot7 overexpressing HFD) have been normalised to control chow and shown as a fold difference. Data is mean ± SEM, n = 8. These animals received either AAV-GFP in control leg (n = 3) or saline injections (n = 5). No differences between the controls were observed.

The effect of Acot7 overexpression on a range of mitochondrial proteins was studied, followed by *ex vivo* flux studies for both glucose and fatty acid metabolism in isolated muscle strips. Overexpression of Acot7 did not alter the content of representative protein components of the mitochondrial electron transport chain or the integral mitochondrial proteins VDAC and UCP3 (Fig. 4).

**Figure 5:**
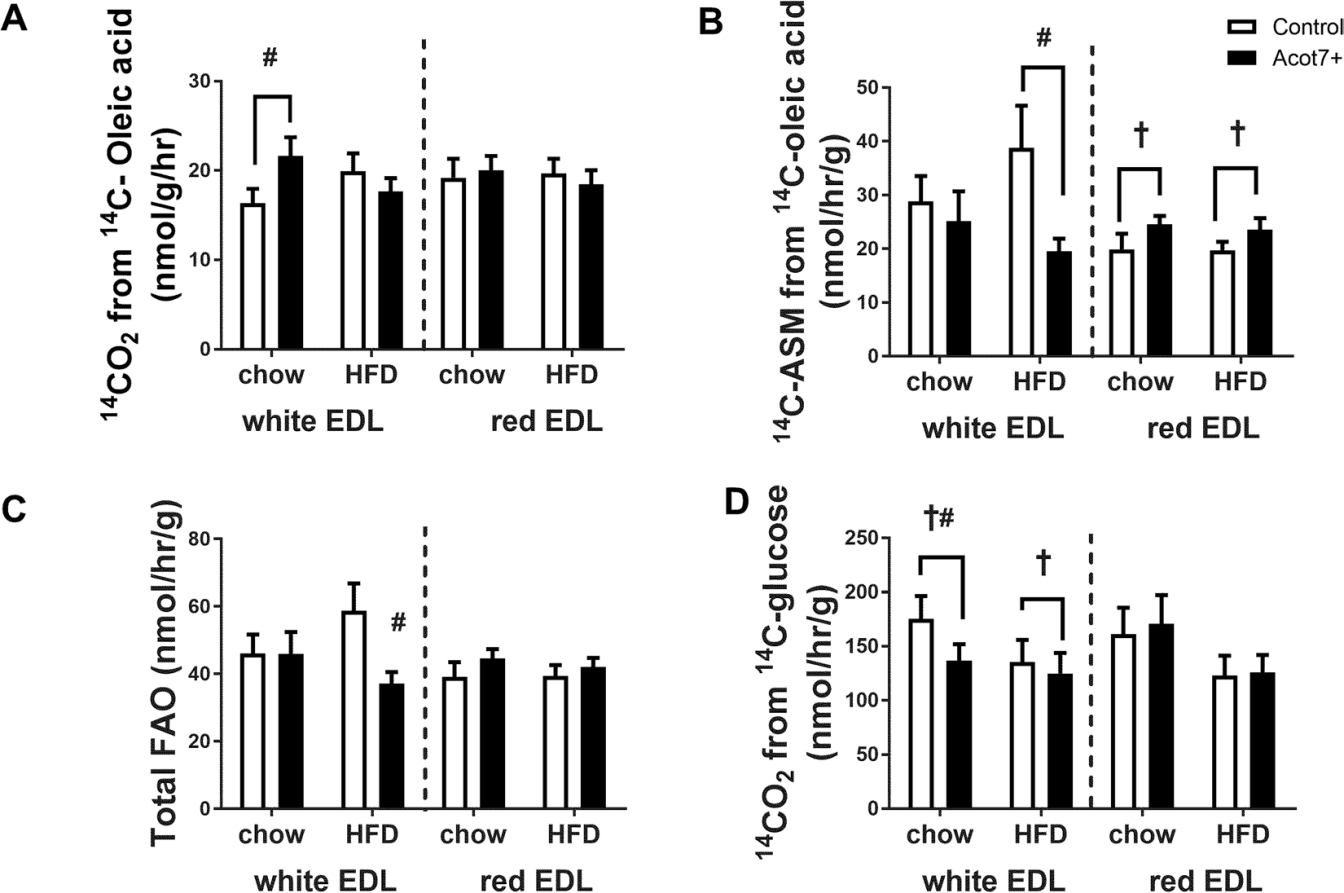
*Ex vivo* effect of AAV9-tMCK-Acot7 on substrate oxidation in EDL (extensor digitorum longus) muscle strips. ^14^C-oleic acid incorporated into ^14^CO_2_ (**A**), ^14^C-oleic acid incorporated into acid soluble metabolites (ASM) (**B**), total fatty acid oxidation (FAO) (C) ^14^C-glucose incorporated into ^14^CO_2_ (**D**) in white and red EDL muscle strips. ^†^p < 0.05, 2-way ANOVA, Acot7; ^#^p < 0.05 post-hoc Holm and Sidak test. Data is mean ± SEM (n = 10–13).

The EDL muscle consists of white (white Type IIb fibres) and red (mixture of red Type IIa, Type IIx fibres) EDL strips, which were incubated *ex vivo*^28,29^. The red and white EDL muscle bundles were stripped from tendon to tendon from control and Acot7 overexpressing legs of chow and HFD rats and were separated out for individual incubations. Overexpression of Acot7 increased thioesterase activity from 0.1 µmol/min/g to 7.0 µmol/min/g and 3.3 µmol/min/g in white and red EDL, respectively. There was no significant effect of the HFD diet on oxidation of [1-^14^C]-oleic acid to ^14^CO_2_ in either red or white EDL muscle strips. There was a small but significant increase in ^14^CO_2_ production in white EDL from chow fed rats with Acot7 overexpression (Fig. 5A). HFD did not alter incomplete β-oxidation as measured by the conversion of [1-^14^C]-oleic acid to acid soluble metabolites (ASM). Acot7 increased incomplete β-oxidation in red EDL of chow and HFD rats, but decreased ASM production from [1-^14^C]-oleic acid in white EDL muscles isolated from HFD rats (Fig. 5B). However, these differences in red and white EDL muscle strips did not translate into any significant differences in the rate of total fatty acid oxidation (^14^CO_2_ production plus conversion to ASM), except for a lower total fatty acid oxidation in white EDL from HFD rats overexpressing Acot7 (Fig. 5C). The white EDL muscle strips overexpressing Acot7 from chow fed animals had a reduced [U-^14^C]-glucose incorporation into ^14^CO_2_ (Fig. 5D). This reduced glucose oxidation and increased complete fat oxidation with overexpression of Acot7 was observed only in white EDL muscle from chow fed animals. Acot7 overexpression had no effect on [U-^14^C]-glucose oxidation in red EDL chow muscle. Red EDL strips isolated from HFD rats appeared to have lower complete [U-^14^C]-glucose oxidation, but this did not reach statistical significance (Fig. 5D). In summary, Acot7 overexpression produced some changes in *ex vivo* substrate oxidation but these changes seemed to be dependent on fibre composition of the muscle strips.

### Effect of Acot7 overexpression on insulin mediated glucose uptake

**Figure 6:**
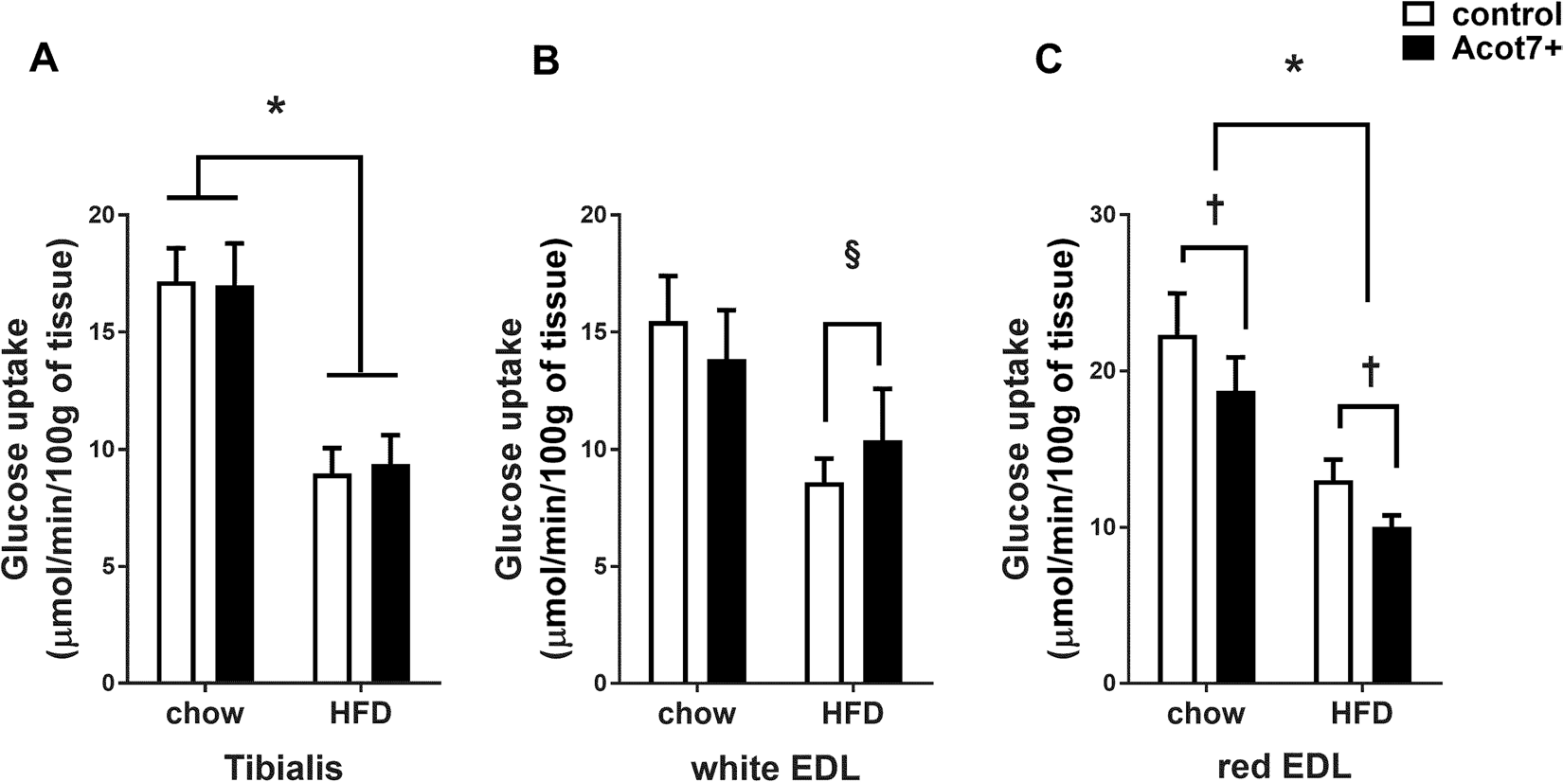
*In vivo* effect of AAV9-tMCK-Acot7 on insulin stimulated glucose uptake in different muscle. Insulin stimulated glucose uptake (Rg’) in tibialis (**A**), white extensor digitorum longus (EDL) (**B**), red extensor digitorum longus (EDL) (**C**). ^§^p = 0.05, *p < 0.01, 2-way ANOVA, diet; ^†^p < 0.05, 2-way ANOVA, Acot7. Data is mean ± SEM (n = 6–8).

Hyperinsulinaemic euglycaemic clamps were performed to determine if there were any effect of increased Acot7 activity and phospholipid changes on insulin action in muscle of control and HFD rats. Insulin action was studied in the tibialis (mixture of red Type IIa, Type IIx and white Type IIb fibres), red EDL (Type IIa, Type IIx fibres) and white EDL (white Type IIb fibres)^28,29^ and red quadriceps muscle, from control muscle beds of one leg and muscle beds of the contralateral leg infected with AAV delivering Acot7. On average, the thioesterase activity of the tibialis and EDL muscle from the AAV-treated leg was increased to 11 µmol/min/g of tissue. The HFD fed rats had increased epididymal fat pad weight and higher basal insulin levels indicating the development of insulin resistance (Table 2). Under hyperinsulinemic/eugylcemic clamp conditions of equally increased insulin levels, the rate of whole body glucose disappearance was significantly less in HFD rats, demonstrating reduced whole-body insulin action (Table 2). All the muscle types examined from HFD animals had impaired insulin stimulated glucose uptake compared to chow fed animals (Fig. 6). In the tibialis muscle (mixture of red Type IIa, Type IIx and white Type IIb fibres) from HFD rats, insulin-stimulated glucose uptake was reduced by approximately 30% and Acot7 overexpression had no effect on glucose uptake in this muscle in chow or HFD rats (Fig. 6A). There was no effect of Acot7 overexpression observed in the white EDL (white Type IIb fibres) (Fig. 6B). Acot7 overexpression produced a small but significant decrease in insulin-stimulated glucose uptake in red EDL (Type IIa, Type IIx fibres) muscle from chow and HFD rats (Fig. 6C). Additionally, a lower level of Acot7 overexpression in Type I fibre rich red quadriceps muscle (4 µmol/min/g of tissue compared to 16 µmol/min/g in tibialis) had no significant effect on insulin-stimulated glucose uptake (Supplementary Fig. S2).

**Table 2:** Characteristics of animals undergoing hyperinsulinaemic/euglycaemic clamp.

	CHOW	HFD	t-Test	2 way ANOVA
Number of animals	8	6		
Body weight (g)	279 ± 13	317 ± 10	#	
Epididymal fat pad (g)	1.2 ± 0.2	2.4 ± 0.3	##	
Subcutaneous fat pad (g)	1.6 ± 0.2	3.0 ± 0.3	##	
Clamped plasma glucose (mM)	7.2 ± 0.2	7.7 ± 0.4		
Basal insulin (mU/L)	38.1 ± 4.7	54.7 ± 5.4	#	**
Final insulin (mU/L)	313 ± 27	306 ± 41		
Basal NEFA (mM)	2.9 ± 0.9	3.4 ± 1		**
Final NEFA (mM)	1.0 ± 0.2	0.9 ± 0 0.4		
Basal TAG (mM)	1.2 ± 0.2	1.0 ± 0.2		**
Final TAG (mM)	0.5 ± 0.1	0.5 ± 0 0.1		
Rate of glucose disappearance	36.6 ± 3.6	25.7 ± 2.5	##	
Rate of glucose infusion	35.3 ± 1.5	24.2 ± 2.6	#	

Data is mean ± SEM; ^#^p < 0.05, ^##^p < 0.01 unpaired t-test between chow and HFD animals, **p < 0.01, 2-way ANOVA, effect of clamp. NEFA = non-esterified fatty acid; TAG = Triglyceride.

## Discussion

Excess lipids from a high fat diet can lead to accumulation of various lipid species thought to impair insulin action in skeletal muscle^30^. All fatty acids (LCFA) are converted to their acyl CoA forms (LCAC) before they are converted into potentially harmful lipid species and the balance between LCFA and LCAC is maintained by Acyl CoA Synthase and thioesterase enzymes. Therefore altering the balance between these two enzyme classes could alter the amount and metabolism of various lipid species. LCAC have been associated with insulin resistance in rodent models^31,32^ and in humans^27^ and so reducing the availability of LCAC by increasing thioesterase activity may improve insulin action in skeletal muscle.

Type II thioesterase, Acot7a and Acot13 have similar structure and substrate specificity for LCFAs^10^ but vary in cellular localisation. Acot7a localises to the cytoplasm while Acot13 localises to the mitochondria. Acot7 is known to regulate fatty acid oxidation^33,34^ and like Acot13 may play a role in insulin action^18^. Acot7 protein expression has been reported to increase in skeletal muscle with high fat feeding (Supplementary Fig. S1)^35^. Therefore, we hypothesised that because of its cellular localisation, increasing Acot7 activity could alter the LCFA/LCAC balance in the cytoplasm to regulate fatty acid metabolism and insulin action in skeletal muscle. The current study examined whether significantly increasing the thioesterase capacity of muscle using an tMCK-AAV9-Acot7, could alter metabolism of fatty acids in a way that might improve glucose utilisation *ex vivo* and *in vivo*.

Total LCAC levels in muscle were increased by HFD but not significantly altered with Acot7 overexpression. However, several clear changes in lipid profile of tibialis muscle were observed in the current study as a result of the HFD treatment or increased Acot7 activity. PE, PC, PG, PS and SM were significantly lower in muscle from HFD rats and the expected increase in triglyceride levels was observed (Table 1). The major effect of Acot7 overexpression on lipid species occurred in muscle of chow fed rats, where higher thioesterase activity decreased PC, PE and PS species to the level observed in HFD muscle, with no further effect of Acot7 overexpression observed in muscle from HFD animals. This lipid profile suggested that PE and PC could be converted to TAGs, especially for lipids containing linoleic acid. This may be achieved by limiting synthesis of PC via the Kennedy pathway. Additionally, Acot7 may increase PE to PC conversion, thus lowering PE levels. In our analysis, individual species for TAG class of lipids were not identified and therefore, this observation could not be confirmed. Further work would be required to estimate the Acot7 mediated changes to lipid pathways in chow animals. High lipid availability, in muscle from HFD fed rats, could reduce Acot7 mediated redirection of certain fatty acids in to specific lipid pathways which would result in limited effects of Acot7 overexpression observed in animals fed a HFD.

Acot7 overexpression produced lipidomic changes across several phospholipid classes in the tibialis muscle of chow-fed muscle similar to those observed with HFD without altering insulin-stimulated glucose uptake. The data show that 56 out of the 68 phospholipids species altered by Acot7 in chow-fed muscle, were reduced towards or equal to phospholipid levels of control HFD muscle, but the lack of any effect on tibialis muscle glucose uptake suggests that it is unlikely that these specific PC, LPC, PE, PG, PS or SM species directly affect insulin action. For example, the results of our study show LPC 18:1 and LPC 18:2 were reduced to similar levels in response to HFD and Acot7 overexpression in chow control muscle. The effect of Acot7 expression to influence the fatty acid composition, as well as the total amount of the different phospholipid classes, suggests that this enzyme may have a specific role in lipid metabolism over and above simple hydrolysis of any LCAC species. Despite these changes insulin action (represented by glucose uptake in a hyperinsulinaemic euglycaemic clamp), remained unaffected in the chow fed muscle. Therefore, our data indicates that LPC 18:1 and LPC 18:2 may not be biomarkers for insulin action in muscle^36^, although they may correlate with insulin resistance in liver^37^. This difference between tissues may help explain the varied results presented in literature on the prognostic potential of plasma levels of LPC species in pre-diabetic and diabetic individuals^38,39^.

Although, previous studies have suggested that the chain length and saturation of fatty acids in phospholipids in the serum/plasma and muscle correlate with insulin resistance^40,41^, it should be noted that not all studies found this correlation^42^. Furthermore, the data obtained in the current and previous studies used different mass spectrometry and chromatographic techniques making it difficult to compare data sets. However, recent studies have extensively estimated the effect of diet or exercise on phospholipids^43–46^, as well as suggested possible biological functions of specific lipid species^47^. Some of these studies show a possible correlation of PC, PE^43,45^, PC/PE ratio^46^, SM or LPC species^37^ with impairment in insulin action in muscle or liver. Conversely, another study found that complete lack of the PE class of lipids did not alter insulin action^48^. Interestingly, changes to phospholipids specifically in the sarcoplasmic reticulum of the skeletal muscle, has been shown to have downstream effects on insulin signalling^6,7^. Clearly, additional work is required to investigate changes in phospholipids and their specific subcellular localisations and how these alterations might modulate insulin action.

The effect of Acot7 overexpression on mitochondrial proteins and function was also studied. AAV-Acot7 transduction in tibialis muscle did not alter mitochondrial proteins or proteins involved in fatty acid metabolism, but Acot7 overexpression did differentially alter substrate oxidation *ex vivo* in white and red EDL from chow fed animals. Lack of Acot7 has been reported to alter overall fatty acid oxidation in the brain but not in the liver and adipose tissue^33,34^. Therefore, any effects of Acot7 on fatty acid oxidation may be tissue-specific and muscle fibre type specific as suggested by our results.

In chow fed animals, Acot7 overexpression in the white EDL strips altered substrate oxidation *ex vivo* by increasing fatty acid oxidation to CO_2_ while decreasing glucose oxidation as might be predicted by the Randle cycle^49^. However these changes were not reflected by any effect on insulin action in white EDL *in vivo*. White EDL strips from HFD animals demonstrated increased fatty acid oxidation and reduced glucose oxidation compared to similar strips from control chow animals. However, this metabolic flexibility did not confer any protection against insulin resistance *in vivo* which is contrary to some other studies^50,51^. On the other hand, Acot7 overexpression in white EDL muscle strips from HFD fed animals showed reduced total fatty acid oxidation as well as the maintenance of reduced glucose oxidation levels. Lower glucose oxidation in white EDL from HFD rats with or without Acot7 overexpression may be due to impaired glucose uptake and glucose transport^52,53^ in white EDL muscle.

In red EDL strips, overexpression of Acot7 increased β-oxidation of oleic acid to ASM, irrespective of diet, which may be attributed to 25% higher β-oxidative capacity in red muscle mitochondria^54^. The observed increase in incomplete fatty acid oxidation may have led to reduced insulin-stimulated glucose uptake in this muscle for both chow and HFD fed animals as proposed by Koves *et al*.^20^. Acylcarnitines, by-products of incomplete fatty acid oxidation, in the muscle has been shown to trigger muscle inflammatory response^55^, oxidative stress^56,57^ and impair Akt phosphorylation^56^ and glucose uptake^56^. An increase in incomplete fatty acid oxidation could impair insulin action mediated by the AMPK pathway^58^. AMPK subunits are known to have fibre type specific expression and this could may contribute towards the differential effects observed in red and white EDL muscle^59,60^. In addition, varied β-hydroxyacyl CoA dehydrogenase expression and regulation in red and white muscle may also contribute to the metabolic differences observed in different fibre types^54,60^, rather than differences in fatty acid uptake^61^.

The thioesterase family of enzymes is only now being systematically investigated, and similar to the ACSL family of enzymes, it may become apparent that certain isoforms of thioesterase have specific subcellular locations that determine their major function in regulating lipid metabolism^3,5,10,25^. In summary, as a cytoplasmic thioesterase, Acot7a may be involved in the regulation of fatty acid incorporation into phospholipids and fibre specific regulation of fatty acid oxidation. Although, increased levels of Acot7 impaired insulin action in red EDL (Type IIa and Type IIx fibres), it was not considered that a similar alteration in all red muscle would change whole body glucose uptake, since only 23% of whole body musculature in rats comprises of red muscle (Type IIa and Type IIx fibres)^28^. The current results do show the potential for studying the role of various thioesterase family members in muscle lipid metabolism and the subsequent evaluation of any impact of these changes on insulin-stimulated glucose metabolism.

## Methods

### Vector Construction and AAV propagation

The human Acot7a was a gift from A/Prof Jade Forwood, Charles Sturt University, Wagga Wagga, Australia. A flag tag was inserted on the N-terminal of the protein by Genscript, U.S.A. and then further subcloned into the pAMCBA vector backbone under the muscle specific promoter tMCK. The plasmid was then used by University of Pennsylvania Vector Core Facility (Philadelphia, PA, USA) to generate 5.28 × 10^13^ genome copies of AAV9-tMCK-FLAG-Acot7.

### Animal maintenance

Young male wistar rats (60 g) were injected in the right tibialis/EDL muscle bundle with 10^11^ genome copies of AAV9-FLAG-Acot7. Saline was injected into the same muscle bundle of the left leg which acted as an internal control. Some of these animals also received a AAV9-FLAG-Acot7 injection in the red quadriceps muscle of the right leg and a saline control injection in the left red quadriceps. Some animals received a AAV9-GFP or saline injections in the left leg and were compared as control when estimating protein levels with western blot. The animals either received a standard chow diet (8% calories from fat, 21% calories from protein, 71% calories from carbohydrate) from Gordon’s Specialty Stock Feeds, NSW, Australia or a high fat diet (HFD; 45% calories from fat (lard), 20% calories from protein, 35% calories from carbohydrates, based on Rodent Diet #D12451 Research Diets, Inc., NJ, USA). The animals were maintained on a 12:12 hr light-dark cycle at 22 ± 0.5 °C, with free access to water. After four weeks, the rats were either used for *ex vivo* or *in vivo* experiments. In pilot studies using AAV-GFP transfection efficiency was estimated to be 60–70% of all fibres in the tibialis/EDL muscle bundle (data not shown). All experimental procedures were approved by the Garvan Institute/St. Vincent’s Hospital Animal Experimentation Ethics Committee and were in accordance with the National Health and Medical Research Council of Australia Guidelines on Animal Experimentation.

### Lipidomics

Lipidomic analyses were performed as described previously^62^. In brief, lipids were extracted from ~25 mg of powdered tibialis muscle according to the method of Matyash *et al*.^63^. An aliquot of each extract was subjected to base hydrolysis and re-extracted to remove ester-linked lipids and improve mass spectrometric analysis of sphingolipids^64^. Both the total lipid and hydrolysed extracts were analysed by mass spectrometery as desribed previously^62^. Lipid species are notated in accordance with recently proposed shorthand by Liebisch *et al*.^65^.

The datasets generated and analysed during the current study has been incorporated in the supplementary data. Any further data can be made available from the corresponding author on request.

### *Ex vivo* muscle strip isolation

Animals on chow or HFD were euthanased and the EDL from each leg was stripped from tendon to tendon with a 27 gauge needle as described previously^66^. The EDL muscle comprises of proximal red muscle (Type I, Type IIa, Type IIx fibres) and distal white muscle (Type IIb fibres)^28,29^. The red and white muscle were divided into two red and two white muscle strips, each weighing 20–30 mg. The muscle strips were then incubated to measure the metabolism of exogenous oleic acid or glucose in an *ex vivo* system as described^67^. Briefly, the strips were incubated for an initial 30 mins in a pre-warmed, pre-gassed (95% O_2_ and 5% CO_2_) KHB buffer (118 mM NaCl, 4.7 mM KCl, 2.5 mM CaCl_2_, 1.2 mM KH_2_PO_4_, 1.2 mM MgSO_4_, 15 mM NaHCO_3_, with 2% fatty acid free BSA, 5 mM glucose and 0.5 mM oleic acid) with agitation at 30 °C. Thereafter, the strips were transferred into vials containing the same buffer with added 1μCi/ml of [^14^C] oleic acid or 1μCi/ml [^14^C] glucose and incubated for 1.5 hours to estimate oleic acid oxidation or glucose oxidation respectively. The muscle strips were snap frozen and used to validate Acot7 overexpression. The NaOH in the centre well was used to determine the [^14^C]-oleic acid or [^14^C]-glucose incorporation into ^14^CO_2_ for oleic acid or glucose oxidation, respectively. For oleic acid oxidation specifically, the media was used to determine [^14^C]-oleic acid incorporation into acid soluble metabolites (ASM) of the β-oxidation and tricarboxylic cycle (TCA). The dpm in the centre well or the media measured by liquid scintillation counting (Beckman LS6000, Beckman Instruments, CA, USA) were further used to calculate the nmoles of oleate converted to ^14^CO_2_ or [^14^C]-ASM/hour/gram of muscle and nmoles of glucose converted to ^14^CO_2_/hour/gram of muscle.

### Hyperinsulinaemic euglycaemic clamp

Animals on chow or HFD had cannulae surgically inserted into the left carotid artery and right jugular vein. After 5–7 days of recovery, rats were semi-fasted overnight (50% of normal daily intake provided at 5 pm) and hyperinsulinaemic euglycaemic clamp was performed as described^66^. Briefly, the cannulae from the concious rats were connected to an infusion line or sampling line. Insulin was infused at a constant rate of 0.3 U/kg/hr and blood glucose monitored via the sampling line. Glucose (30%) was infused to establish a stable blood glucose of 5–6 mM. After a steady state glucose level was achieved, a bolus injection of 50 μCi ^3^[H]-2-deoxy-D-[2,6]-glucose (PerkinElmer, Glen Waverley, Victoria, Australia) and 22.5 μCi U-[^14^C]-Glucose was administered, and blood samples were taken at 2, 5, 10, 15, 20, 30, and 45 min. At the conclusion of the tracer period, rats were given an intravenous bolus of pentobarbital sodium (60 mg/kg) for euthanasia (Nembutal; Abbott Laboratories, Sydney, Australia) and the tibialis (10% Type IIa, 26% Type IId/x and 61% Type IIb), red EDL (4% Type I, 27% Type IIa, 69% Type IId/x), white EDL (~95% Type IIb)^28^ and red quadricep (~95% Type I) muscles rapidly dissected and freeze clamped for subsequent determination of insulin-stimulated glucose uptake.

### Thioesterase activity assay

Tissue was homogenised in a buffer (20 mM Tri-HCl (pH 8.0), 37 mM NaCl, 1 mM EDTA, 10% Glycerol, 0.5% Triton X-100). The homogenate was centrifuged (10,000 g, 4 °C 5 mins) and 20 µl of the supernatant was added to 500 µl of assay buffer (30 °C) (100 mM KCl, 20 mM HEPES, 0.6 mM 5,5′-dithiobis-2-nitrobenzoic acid (DTNB) pH 7.5). The reaction was started by addition of 2.5 mM palmitoyl-CoA and absorbance monitored for 5–10 minutes at 412 nm. An extinction coefficient of 13,600 M^−1^ cm^−1^ was used to calculate the thioesterase activity^26^.

### Acyl CoA synthase activity assay

Acyl CoA synthase activity assay was estimated as described^27^. Briefly, the tissue was homogenised in homogenising buffer (20 mM Tris HCl (pH 8.0), 37 mM NaCl, 1 mM EDTA, 10% Glycerol, 0.5% Triton X-100) and centrifuged (16,200 g, 4 °C, 5 mins). Homogenate (50 µl) was added to 155 µl of warm reaction buffer (300 mM Tris·HCl and 150 mM MgCl_2_ (pH 7.4), 20 µl Triton X-100, 20 mM ATP, 2.25 mM glutathione, and 0.2 mM CoASH 200 μM [1-^14^C]-palmitate, 0.01 μCi/assay) and 45 µl water. The reaction was started with pre-warmed homogenate (50 µl) and terminated with Dole’s reagent (40 isopropanol-10 hexane-1 0.5 M H_2_SO_4_) at 0, 2, 5, or 10 min for each sample. A phase seperation was obtained with the addition of water and hexane mixture^68^ and the lower layer was washed twice with hexane and used to count [^14^C] palmitoyl CoA by liquid scintillation counting (Beckman LS6000, Beckman Instruments, CA, USA). ACSL activity was expressed as µmol [^14^C] palmitoyl CoA formed/minute/gram of wet weight tissue.

### Western blotting

Protein quantification was carried out by immunoblotting using 1:1000 dilutions of antibodies for Acot7 (Abcam85151), ACSL1 (Cell Signalling 4047), Mitochondrial complex (Mitosciences601), VDAC (Cell SignalingS4866), UCP3 (Abcam4866) as described^22^. Briefly, lysates were prepared in Laemmli, resolved by SDS-PAGE electrophoresis on either 7.5% or 10% gels and proteins transferred to PVDF membrane (Hybond-P). Membranes were blocked in 2% BSA-Tris buffered saline with 0.1% Tween and incubated with primary antibody. After incubation with the appropriate secondary anitbody bands were visualised by chemiluminescence (Western lightning, Perkin Elmer) with exposure to film (Super RX Fuji Photo Film).

### Long Chain Acyl CoA determination

LCAC content was measured fluorometrically as described^69^. Muscle tissue was homogenised in 10% TCA and centrifuged (800 g, 10 mins, 4 °C).The pellet was washed with 1 ml diethyether and then twice with water. The final pellet was resuspended in DTT and hydrolysed with KOH at 55°C for 10 mins. The sample was then neutralised in the presence of a KH_2_PO_4_ buffer and centrifuged (16,200 g for 10 mins) to remove any particulate matter. A sample of the extract was added to reaction buffer (50 mM KH_2_PO_4_ pH7, 13 µM α-ketoglutarate, 2 µM NAD^+^) and fluorecence produced by NADH was measured using a fluorometer after the addition of α-ketoglutarate dehydrogenase. The amount of LCAC was calculated as nmoles CoA/mg protein using a CoA standard curve.

### Statistical Analysis

All data are reported as mean ± SEM. Data were analysed by 2-way ANOVA repeated measures, as comparisons were between overexpressing Acot7 muscle and saline control muscle from the same animal. The 2-way ANOVA was followed by post-hoc analysis. Sidak or the Sidak and Holm test post hoc test was performed as appropriate. Statistical significance was defined as p < 0.05.

## Electronic supplementary material

Supplemental data
