# Supplementary material for: Increasing Acyl CoA thioesterase activity alters phospholipid profile without effect on insulin action in skeletal muscle of rats
Source: Sci Rep. 2018 Sep 18;8:13967. doi: 10.1038/s41598-018-32354-w (PMC6143561; doi:10.1038/s41598-018-32354-w)

Increasing Acyl CoA thioesterase 7 activity alters phospholipid profile without effect on insulin action in skeletal muscle of rats.

Ishita Bakshi[1], Simon H.J. Brown[2], Amanda E Brandon[1,3], Eurwin Suryana[1], Todd W. Mitchell[2], Nigel Turner[4], and Gregory J Cooney[1,3*]

[1]Diabetes and Metabolism Division, Garvan Institute, Sydney, Australia. [2]School of Biological Sciences, University of Wollongong, Wollongong, Australia. [3]Sydney Medical School, Charles Perkins Centre, University of Sydney, Sydney, Australia. [4]Department of Pharmacology, University of New South Wales, Sydney, Australia.

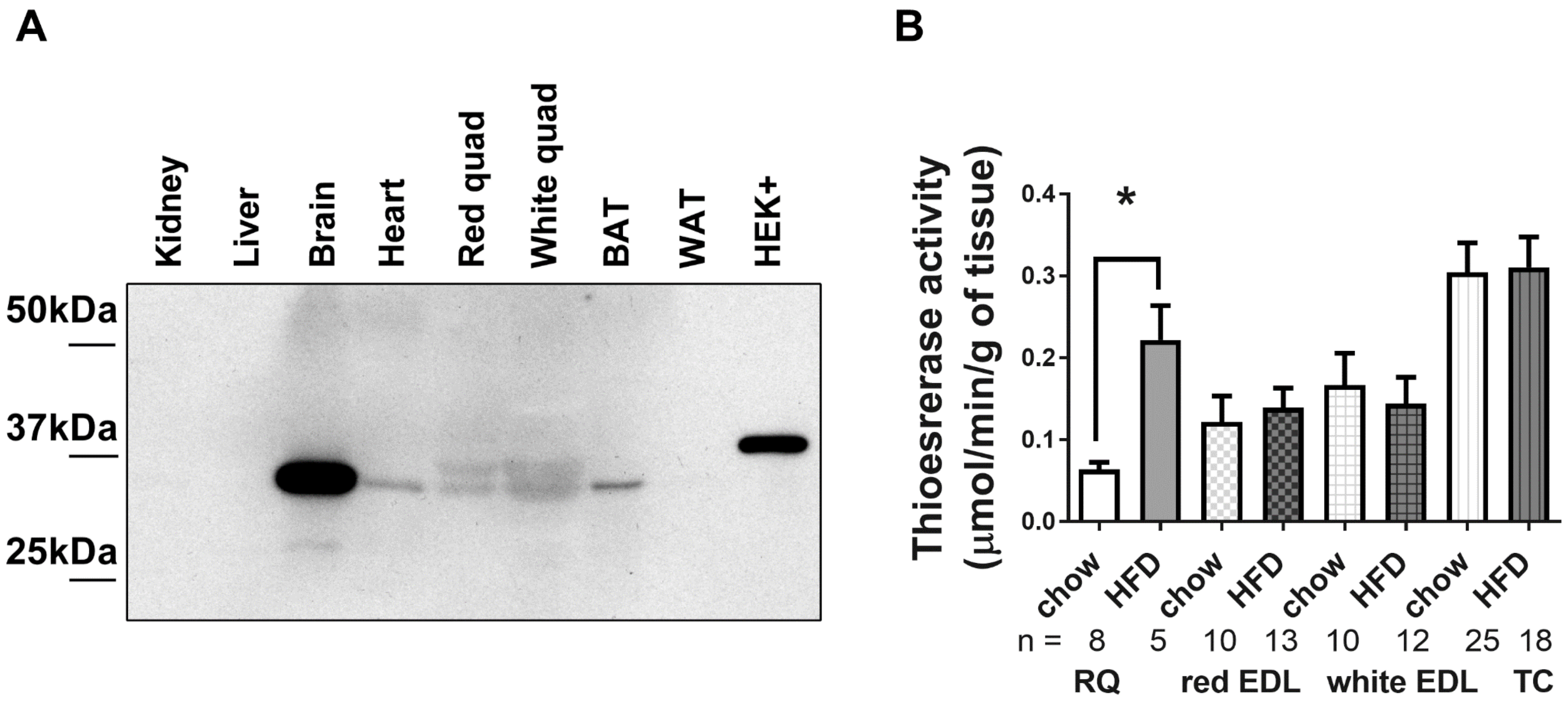

***Supplementary Figure 1*. Tissue distribution profile of Acot7 and effect of HFD on thioesterase activity in different muscles.** *Tissue distribution of Acot7 with the tissue indicated at the top of each lane. HEK+ indicates HEK cells transfected with flag tagged Acot7 (A). Thioesterase activity was measured in different skeletal muscles (RQ, red quadricep; red EDL, red extensor digitorum longus; white EDL, white extensor digitorum longus; TC tibialis cranialis) of chow and high fat fed rats (B). n=5-25. Data is mean ± SEM, *p<0.05, unpaired t-test.*

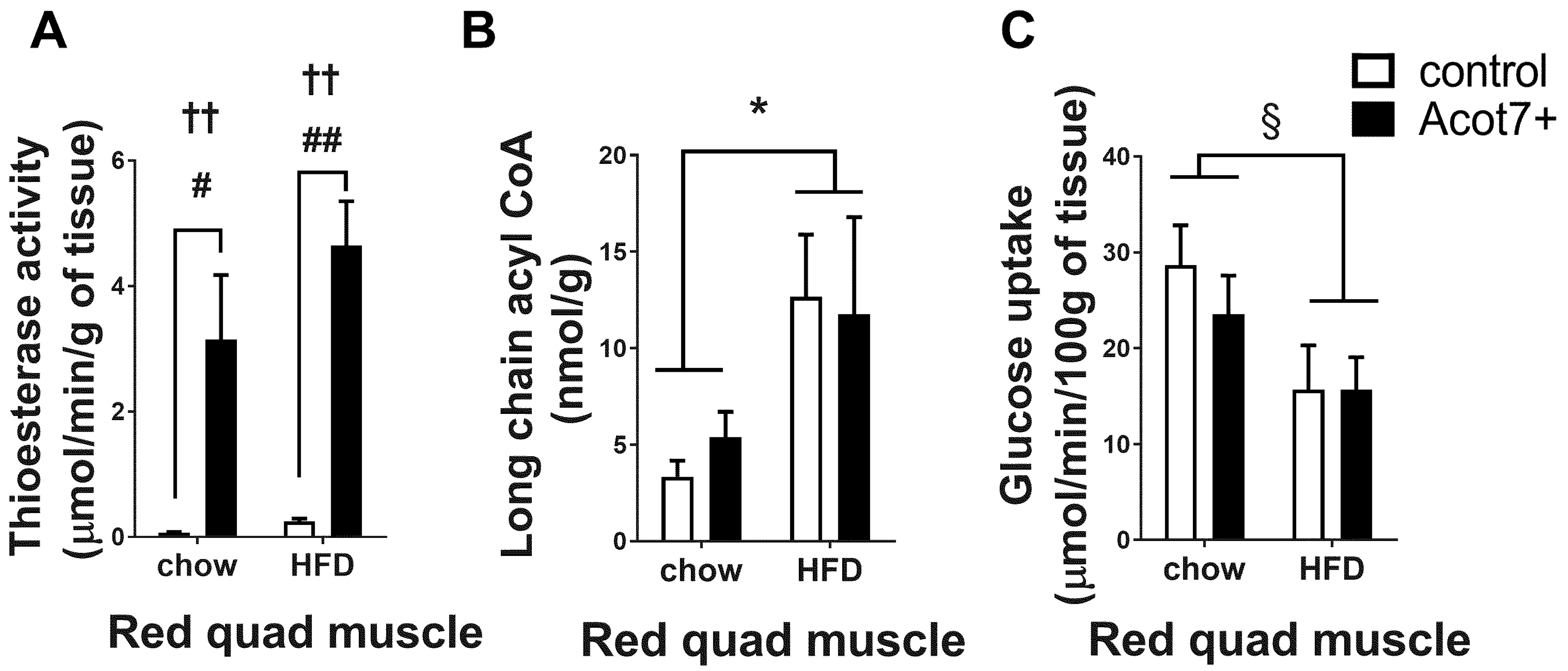

Supplementary Figure 2. Effect of Acot7 on red quadricep muscle. *Thioesterase activity (A), Long chain acyl CoA (B) and glucose uptake (C) in the red quadricep muscle from chow and HFD fed animals. *p<0.05, 2-way ANOVA, effect of diet; ††p<0.01 2-way ANOVA, effect of Acot7; #p<0.05, ##p<0.01 post hoc Holm and Sidak test, §p<0.05 Fisher test.*

Table 1. Lipid species altered by diet and or Acot7.

Lipid class	Lipid species	Lipid levels detected (nmol/g)				2-way ANOVA		
		Control Chow	Acot7 Chow	Control HFD	Acot7 HFD	Interaction	Diet	Acot7
LPC	18:1	17.6 ± 1	13.8 ± 0.9#	13.6 ± 1	11.6 ± 0.6		**	*
LPC	18:2	27 ± 2	21.3 ± 1.1	18.3 ± 2.2	14.4 ± 1.4		**	*
TAG	48:3	6.7 ± 0.6	13.2 ± 2.2#	5.7 ± 1.5	7.1 ± 0.7		*	*
Lipids altered with Acot7 overexpression alone								
Cer	16:0	3.1 ± 0.3	4.2 ± 0.3#	3 ± 0.1	3.2 ± 0.3			*
Cer	22:0	1.9 ± 0.2	1.6 ± 0.1	1.9 ± 0.1	1.4 ± 0.1			*
LPC	22:4	4.7 ± 0.6	2.7 ± 0.7##	4 ± 0.1	4.8 ± 0.4	**		
SM	23:1	12.9 ± 1.8	7.2 ± 1.3#	7.6 ± 1.4	9 ± 0.8	*		
SM	23:2	3.1 ± 1.4	0.1 ± 0.1#	0	0.5 ± 0.4	*		
SM	26:2	0.3 ± 0.3	2.1 ± 0.9#	0	0.4 ± 0.2			*
PG	18:1_18:1	4.9 ± 0.4	3.5 ± 0.4##	3.6 ± 0.4	3.2 ± 0.4			**
PG	18:1_18:2	1.2 ± 0.5	0.3 ± 0.2#	0.9 ± 0.2	1.6 ± 0.2	**		
TAG	56:8	1.9 ± 0.8	5.2 ± 0.9#	3.2 ± 0.6	3.9 ± 0.3			*
TAG	58:8	1.2 ± 0.3	2.8 ± 0.7	1 ± 0.1	2.7 ± 0.8			*
TAG	58:9	0.1 ± 0.1	1 ± 0.5	0	0.6 ± 0.3			*

CL	72:5	66 ± 16.4	72.4 ± 8.5	111.8 ± 12.4	66.9 ± 10.1#	*		
CL	72:8	1446 ± 86	970 ± 72#	1163.7 ± 159	1079.7 ± 86.4			*
CL	76:11	48.3 ± 7.5	15.7 ± 3.6#	26.1 ± 5.9	39.1 ± 9	**		
Different lipid species content that are altered by Acot7 overexpression. Data is mean ± SEM, nmol/g. 2-way ANOVA * p<0.05, ** p<0.01 was followed by post hoc Sidak test was performed, #p<0.05, ## p<0.01. n=6								

Table 2. Effect of diet and Acot7 overexpression on Phosphatidylethanolamine species.

PE Species	Control Chow	Acot7 Chow	Control HFD	Acot7 HFD	2-way ANOVA

					Interaction	Diet	Acot7
PE (34:1) 16:0_18:1	32.3±2.4	26.2±1.6	28.5±2.4	24.8±1.4			*
PE (34:1) 16:1_18:0	6.9±0.6	4.3±0.4##	2.1±0.7	2.6±0.2	**	**	*
PE (34:2) 16:0_18:2	69±4.5	48.8±1.1##	47.7±6.5	36.2±3.1		**	**
PE (34:2) 16:1_18:1	4.7±0.5	1.7±0.4##	1±0.5	0.2±0.2	**	**	**
PE (36:0) 18:0_18:0	6.5±0.1	5.5±0.4	4.3±0.3	4.6±0.3		**	
PE (36:1) 18:0_18:1	53.3±2.8	37.2±1.4##	37.8±2.1	35.6±2.2	*	**	**
PE (36:2) 18:0_18:2	357.9±17.1	230.2±8.3##	229.1±27.5	184.2±16.1	*	**	**
PE (36:2) 18:1_18:1	17.2±0.8	10.5±0.5##	8.6±0.5	7.9±0.4	**	**	**
PE (36:3) 16:0_20:3	6.3±0.4	5.9±0.7	4.8±0.5	3.6±0.3		**	
PE (36:3) 18:1_18:2	127.5±6.8	77.5±4.2##	57.4±5.7	49.1±4	**	**	**
PE (36:4) 16:0_20:4	80.1±3.8	71.5±3.3	102.1±5.6	110.5±5		**	
PE (36:4) 18:2_18:2	57.7±3.9	35.3±1.3##	27.9±4.6	25.6±1.6	**	**	**
PE (36:5) 16:0_20:5	3.2±0.4	1±0.3##	0	0	**	**	**
PE (36:5) 16:1_20:4	7.7±0.6	5.5±0.3##	4.2±0.3	3.6±0.4	*	**	**
PE (38:3) 18:0_20:3	100.4±4.8	77.8±3.4##	112.7±5.9	116.3±6.6		**	
PE (38:4) 16:0_22:4	26.9±1.1	20.9±1.1##	45.8±1.9	45.3±1.7		**	
PE (38:4) 18:0_20:4	767±31.7	633.2±28#	1037.3±58.7	1097.5±64.8		**	
PE (38:4) 18:1_20:3	4.9±0.4	3.2±0.4#	1.5±0.4	1.3±0.3		**	*
PE (38:5) 16:0_22:5	56.9±1.6	43.4±2##	90.8±8.9	94.4±7.8		**	
PE (38:5) 18:0_20:5	45.4±2.3	21.5±1.8##	5.5±0.7	4.6±0.7	**	**	**
PE (38:5) 18:1_20:4	142.6±7.3	104.2±4.4##	113.6±8.1	111.2±6.7	*		*
PE (38:5) 18:2_20:3	3.4±0.4	1.1±0.6##	0	0.2±0.2	**	**	*
PE (38:6) 16:0_22:6	345.1±13.8	265.8±13.5##	143.7±12.6	152±12	**	**	*
PE (38:6) 16:1_22:5	12±0.7	10.5±0.5#	10.3±0.8	11.8±0.7	*		
PE (38:6) 18:1_20:5	9.4±0.9	3.8±1##	0	0	**	**	**

PE (38:6) 18:2_20:4	45.6±1.5	35.3±2.2#	42.9±2.5	46.8±2.9	*		
PE (38:7) 16:1_22:6	16.5±0.8	10.2±0.7##	6±0.7	5.6±0.4	**	**	**
PE (40:4) 18:0_22:4	45.1±2.4	29.6±1.4##	93±6.2	92.8±6.1		**	
PE (40:5) 18:0_22:5	291.3±14	212.6±8.3##	455.4±46.9	487.7±42.5		**	
PE (40:5) 18:1_22:4	22.9±1.5	14.2±1.5##	15.2±0.9	14.6±0.9	*	**	*
PE (40:6) 18:0_22:6	1591.3±73	1176.9±54.5##	774.9±54.5	810.5±61.9	**	**	*
PE (40:6) 18:1_22:5	48.6±2.9	29.6±2##	45.4±3.8	45.7±2.8	**		**
PE (40:7) 18:1_22:6	407±29.1	253.3±16.9##	136.4±8	133.6±8.1	**	**	**
PE (40:8) 18:2_22:6	180±9.3	122.1±6.5##	66.3±3.5	63.9±4.6	**	**	**
PE (42:10) 20:4_22:6	7.2±0.9	4.8±0.4##	3.3±0.3	3.1±0.3	*	**	*
PE (42:9) 20:3_22:6	21.6±1.8	5.5±3.5##	3.8±2.5	4.9±2.2	*	**	*
PE (O_34:1) O_18:1_16:0	13.9±1.2	11.9±0.9	14.2±1	11±2.4			
PE (O_34:2) O_16:1_18:1	4.3±0.5	4.3±0.5	4.7±0.4	3.9±0.2			
PE (O_34:2) O_18:2_16:0	9±0.9	8.2±1.1	8.1±0.8	7.2±0.7			
PE (O_34:3) O_16:1_18:2	1.2±1.2	0	2.8±1.3	3.9±1.3			
PE (O_36:2) O_18:1_18:1	37.6±3.5	25.2±1.3##	16.2±1.1	12.9±1.1	*	**	**
PE (O_36:3) O_18:1_18:2	6±2.7	5.9±1.9	10.2±1.2	8.2±1.7			
PE (O_36:4) O_16:1_20:3	7.9±1.6	7.1±1.8	9.4±0.5	8.9±0.8			
PE (O_36:5) O_16:1_20:4	31.4±1.2	26.1±1.9	41.7±2.8	43.4±2.9		**	
PE (O_38:4) O_18:0_20:4	56.4±3.6	44.3±1.9	49±7.9	40.9±2.8			*
PE (O_38:4) O_18:1_20:3	10.7±2.3	6.2±2.8	13.2±4.6	24.2±2.5##	**	*	
PE (O_38:5) O_16:1_22:4	22.3±1.8	19.3±0.8	48±3.9	49±2.8		**	
PE (O_38:5) O_18:1_20:4	44.8±2.9	39.6±3.2	63.8±2.1	74.1±3.6#	*	**	
PE (O_38:5) O_18:2_20:3	1.7±0.4	1±0.5	0.4±0.2	0.9±0.3			
PE (O_38:6) O_16:1_22:5	88.3±3.6	76.7±3.3	106.1±5.8	117.2±5.1		**	
PE (O_38:6) O_18:1_20:5	2.1±0.2	2±0.1	0	0.1±0.1		**	
PE (O_38:6) O_18:2_20:4	36.8±1.6	34.2±3.3	25.6±1.6	28±2		**	

PE (O_38:7) O_16:1_22:6	173.8±3.3	153±8.4	108.1±4.5	121.6±8.2	*	**	
PE (O_40:5) O_18:1_22:4	46.6±3.7	36.4±2.4	56.3±2.3	64.4±2.4	*	**	
PE (O_40:6) O_18:0_22:6	27.9±2.6	15.6±1##	1.3±0.5	1.4±0.4	**	**	**
PE (O_40:6) O_18:1_22:5	108.3±4.1	87.1±5.4#	81.1±3	91.9±5.6	*	*	
PE (O_40:6) O_18:2_22:4	20.8±1.4	12.7±1##	11	10.1±0.4	**	**	**
PE (O_40:7) O_18:1_22:6	77.2±3.8	70.1±5.6	57.9±3.1	67.2±5.3			
PE (O_40:7) O_18:2_22:5	30.2±2.1	23.4±1.5#	18.4±1.4	19.3±0.8	*	**	
PE (O_40:8) O_18:2_22:6	91.1±4.2	70.2±5.1##	30.1±1.2	34.2±2.2	**	**	*

Phosphatidylethanolamine content in each group. Data is mean ± SEM, nmol/g. *p<0.05, **p<0.01 2-way ANOVA followed by #p<0.05, ##p<0.01, post hoc test Holm and Sidak or Sidak was performed. N=6

Table 3. Effect of diet and Acot7 overexpression in Phosphatidylcholine species.

PC Species	Control Chow	Acot7 Chow	Control HFD	Acot7 HFD	2-way ANOVA		
					Interaction	Diet	Acot7
PC (32:0) 16:0_16:0	239.3±20.4	212.4±11.8	243.3±5.4	250.9±19.1			
PC (32:1) 16:0_16:1	149.9±11	103.5±8.8#	91.9±4.9	77.5±4.3		**	*
PC (34:0) 16:0_18:0	18.1±2.3	16±1.5	35.3±0.6	30.4±4.2		**	
PC (34:1) 16:0_18:1	1566.4±100.3	1270.8±92.4	1211.4±49.4	1293.2±67.8		**	
PC (34:1) 16:1_18:0	17.6±1.2	13.7±0.9	11±1.3	9.4±0.6		**	*
PC (34:2) 16:0_18:2	3236.1±229.1	2429.6±137.3#	1971.5±196.9	1598.6±127.8		**	*
PC (34:2) 16:1_18:1	90.7±4.8	56.7±5.4##	35±1.5	26.7±1.4	*	**	**
PC (34:3) 16:0_18:3	68.4±4.6	46.1±2.7##	22.6±1.2	18.1±1.1	*	**	**
PC (34:3) 16:1_18:2	42.9±2.1	35.4±3.5	23.8±1.4	26±2.6		**	
PC (36:0) 18:0_18:0	97.8±4.5	89.5±7.8	53.2±3.2	65.2±5		**	
PC (36:1) 18:0_18:1	165.1±13.1	134.6±5.7	152.8±5.8	144.6±13			
PC (36:2) 18:0_18:2	964.4±62.8	709.2±47.9#	647.4±63.5	553.2±61.9		**	**
PC (36:2) 18:1_18:1	322.1±12.5	209.5±15.3##	156.7±5.4	142.8±7	**	**	**
PC (36:3) 16:0_20:3	211.3±9.2	177.7±11.1	116.9±8.9	106.5±8.1		**	
PC (36:3) 18:1_18:2	668.3±27.6	417.5±22##	290.9±23.5	211.7±22.4	**	**	**
PC (36:4) 16:0_20:4	2942.7±173.4	2504.5±115.4	4669.8±233	4854.6±242		**	
PC (36:4) 18:2_18:2	183.8±17	119±4.4##	69.2±8.1	48.3±5.6	*	**	**
PC (36:5) 16:0_20:5	181.7±12.6	105.4±5.4##	32.6±3.5	24.5±1.3	**	**	**
PC (36:5) 16:1_20:4	43.7±2.7	30.7±2.4#	51.5±4.6	44±4.5		*	*
PC (38:3) 18:0_20:3	79.2±7	61.2±4.9	35.5±5.1	27.9±1.7		**	*
PC (38:4) 16:0_22:4	38.6±3.1	28.5±1.8	110.2±6.2	110.2±8.9		**	
PC (38:4) 18:0_20:4	901.7±58.5	779.1±42.9	1675.4±107.8	1733.5±163.2		**	
PC (38:4) 18:1_20:3	33.2±2.3	22.7±1.7##	8.1±1.4	10.5±1.2	**	**	*
PC (38:5) 16:0_22:5	321.6±28.8	285.6±15.1	375.9±27.8	435.9±34.2		**	
PC (38:5) 18:0_20:5	18.5±1.3	11.4±0.8##	0.8±0.8	0	**	**	**
PC (38:5) 18:1_20:4	416.7±15.9	306.4±16.3##	436.6±23.7	400.9±24.2		**	*

PC (38:5) 18:2_20:3	14±0.9	9.7±1.2$^{\#}$	3.8±1.3	2.9±1.1		**	*
PC (38:6) 16:0_22:6	1499.6±94.1	1360.7±68.5$^{\#}$	588.8±55	669.8±61.9		**	
PC (38:6) 18:1_20:5	26.6±2.2	12.7±2.1$^{\#\#}$	0	0	**	**	**
PC (38:6) 18:2_20:4	156.7±15.8	120.6±4.6	182.4±6.3	166.6±13		**	
PC (40:4) 18:0_22:4	14.7±1.4	8.6±1.8$^{\#}$	29.6±1.5	29.3±2.8		**	
PC (40:5) 18:0_22:5	83.5±6.1	71.4±3.2	103.4±8	105.9±11.1		*	
PC (40:6) 18:0_22:6	261.5±14.1	228.1±9.1	115.1±12.2	123.6±14.4		**	
PC (40:6) 18:1_22:5	29.5±2.7	19.7±1.4$^{\#}$	19.4±2.5	21.3±1.6	*		
PC (40:7) 18:1_22:6	150.6±8.7	105.2±5.7$^{\#\#}$	46.5±3.4	45.2±3.9	**	**	**
PC (40:8) 18:2_22:6	99±7.6	78.4±5.1$^{\#}$	39±3	39.3±2.8		**	
PC (O_32:0) O_16:0_16:0	26.9±2.4	24.6±1	17.2±0.4	16.4±1.1		**	
PC (O_32:1) O_16:1_16:0	11.5±0.8	10.3±0.6	12±0.3	12.5±1.2			
PC (O_34:1) O_18:1_16:0	65.9±3.1	52.2±3.1$^{\#}$	36.6±0.9	35.8±2.6		**	
PC (O_36:2) O_18:0_18:2	108.6±8.2	74.8±5.4$^{\#\#}$	34.2±2.4	29.4±2.1		**	*
PC (O_36:4) O_16:0_20:4	37.5±2.8	27.9±1.2$^{\#}$	24.4±1.1	25.9±1.5	*	**	
PC (O_36:5) O_16:1_20:4	44.3±3.9	43.1±5.3	64.8±2	78.8±6.8		**	
PC (O_38:4) O_18:0_20:4	82.3±6.5	63.5±3.4	74.5±3.6	71.1±4.9			
PC (O_38:5) O_18:1_20:4	33.4±2.3	25.5±1.4	33.7±0.9	35.7±2.7	*	*	

Phosphatidylcholine content in each group. Data is mean ± SEM, nmol/g. *p<0.05, **p<0.01 2-way ANOVA followed by $^{\#}$p<0.05, $^{\#\#}$p<0.01 post hoc Sidak test was performed. N=6

Table 4. Effect of diet and Acot7 overexpression on DAG species.

DAG species	Control Chow	Acot7 Chow	Control HFD	Acot7 HFD	2-way ANOVA		
					Interaction	Diet	Acot7
DAG (32:0) 16:0_16:0	90.2±4	98.7±13.9	112.5±6.3	120.6±11.8		**	

DAG (32:1) 16:0_16:1	14.8±1.1	13.2±1.6	10.7±0.9	10.1±1		*	
DAG (34:0) 16:0 _18:0	56.6±2.6	68.6±12.1	90.1±4.8	87.7±9.4		**	
DAG (34:1) 16:0 _18:1	317.9±14.3	273.9±14.4	279.7±11.8	265.1±21.6			
DAG (34:2) 16:0 _18:2	100.6±5.3	92.3±5.5	112.1±7.3	110.3±7.9			
DAG (34:2) 16:1 _18:1	19.6±1.8	19.1±1.6	11.2±1	11.3±1.1		**	
DAG (34:3) 16:1_18:2	3.7±1.7	5±1.6	0	2.7±1.3		*	
DAG (36:0) 18:0 _18:0	22.3±1.4	26.9±3.6	27.3±1.9	28.2±2.8			
DAG (36:1) 18:0 _18:1	32.4±3.9	32.5±1.5	68.6±5.3	55.3±5.7		**	
DAG (36:2) 18:0 _18:2	27.8±3.1	27±1.8	46.9±3.6	38.5±3.3		**	
DAG (36:2) 18:1 _18:1	185.4±13	161.5±9.2	170.6±13.7	151.2±16.4			
DAG (36:3) 18:1 _18:2	144.3±12.4	134.9±7	149.4±12.9	138.1±16.2			
DAG (36:4) 16:0 _20:4	6.2±1.5	7±1.5	18.6±2.2	18.6±1.6		**	
DAG (36:4) 18:2 _18:2	22.7±2.9	19.3±1.4	23.7±2.4	22±2.8			
DAG (38:4) 18:0 _20:4	28±4.7	31±3.4	39.8±3.3	44.6±4.8		*	
DAG (38:4) 18:1 _20:3	0.9±0.9	0	2±1.3	0.8±0.8			
DAG (38:5) 18:1 _20:4	8.2±3.8	10.7±2.3	18.4±2.6	16.3±1.8		*	
DAG (38:6) 18:2 _20:4	0	0	4.2±1.4	4.1±1.4		**	
DAG (40:5) 18:0 _22:5	2.5±1.8	1±1	10.7±2.6	12.2±2.7		**	
DAG (40:6) 18:0 _22:6	19.2±3.9	21.9±4.3	14.5±3.6	18.1±2.5			
Diacylglyceride content in each group. Data is mean ± SEM, nmol/g. *p<0.05, **p<0.01 2-way ANOVA followed by $^{\#}$p<0.05, $^{\#\#}$p<0.01 post hoc Sidak test was performed. N=6.							

Table 5. Effect of diet and Acot7 overexpression on TAG species.

TAG species	Control Chow	Acot7 Chow	Control HFD	Acot7 HFD	2-way ANOVA		
					Interaction	Diet	Acot7
TAG 48:0	45.6±4	71.7±4.2	100±13.9	103.8±8.8		**	

TAG 48:1	75.4±6.5	124.8±18.3	110.4±21.3	113.2±9.5			
TAG 48:2	45.3±4.6	79.4±12	55.5±11.9	60.7±5.6			
TAG 48:3	6.7±0.6	13.2±2.2$^{\#}$	5.7±1.5	7.1±0.7		*	*
TAG 50:0	24.1±1.6	34.5±3	80.5±9.2	84±7.8		**	
TAG 50:1	266.2±24.2	382.2±48.8	597.8±97.2	635±43.6		**	
TAG 50:2	306.4±31.1	491.2±68.8	578.7±106.5	612.3±45		**	
TAG 50:3	124.8±14.1	216.3±34.6	182.1±37.8	195.1±14.1			
TAG 50:4	24.3±3.1	43.7±7	28.1±6.4	30.8±2			
TAG 52:0	4.4±0.7	8.1±1.2	21.2±2	22.6±2.5		**	
TAG 52:1	77.7±7	100.8±12	274.2±41.3	283.6±21.4		**	
TAG 52:2	453.8±48.4	653.9±91.9	1007.5±174.9	1068.4±79.7		**	
TAG 52:3	442.4±55.2	703.7±109.9	908±166.8	991.1±75.1		**	
TAG 52:4	170.5±22.7	290.8±48.8	282.3±54.5	308.4±23.7			
TAG 52:5	37±5.3	66.5±10.9	36±7.5	40.5±3.5		*	
TAG 54:0	0	0.6±0.4	1.8±0.4	1±0.4	*	*	
TAG 54:1	5.7±0.4	7.2±1	30.2±4.2	30.7±3		**	
TAG 54:2	55.5±7	77.8±11.2	208.4±33.5	215.9±18.7		**	
TAG 54:3	224.2±27.6	339.7±54.5	522.4±100.3	552.9±38.3		**	
TAG 54:4	241.1±32.6	376.3±63.6	476.2±90.6	511.8±33.7		**	
TAG 54:5	115.1±16.4	189.7±31.8	193.4±34.8	212.7±15.4		*	
TAG 54:6	47±8.8	66.1±10.3	49.3±9.1	52.8±4.6			
TAG 56:4	6.4±0.8	9.5±1.5	17.8±2.9	19.3±1.5		**	
TAG 56:5	7.4±1.4	11.5±1.9	26.1±3.6	28.3±2.1		**	
TAG 56:6	22.8±5.7	22.7±3.5	32.4±5	36.8±3		**	
TAG 56:7	10±1.5	16.9±2.6	16.7±2.2	19±1.3		*	
TAG 56:8	1.9±0.8	5.2±0.9$^{\#}$	3.2±0.6	3.9±0.3			*
TAG 58:8	1.2±0.3	2.8±0.7	1±0.1	2.7±0.8			*
TAG 58:9	0.1±0.1	1±0.5	0	0.6±0.3			*
Triglyceride content in each group. Data is mean ± SEM, nmol/g. *p<0.05, **p<0.01 2-way ANOVA followed by $^{\#}$p<0.05, $^{\#\#}$p<0.01 post							

hoc Sidak test was performed. N=6.

Table 6. Effect of diet and Acot7 overexpression on Sphingomyelin species.

SM species	Control Chow	Acot7 Chow	Control HFD	Acot7 HFD	2-way ANOVA		
					Interaction	Diet	Acot7
SM 13:0	0	0.1±0.1	0.1±0.1	0.5±0.3			
SM 14:0	0.2±0.2	0	0.3±0.2	0.2±0.2			
SM 15:0	1.4±0.4	1.4±0.4	1±0.2	0.9±0.3			
SM 16:0	115.2±9.7	105.5±9.9	96.4±5.1	107.3±6.1			
SM 16:1	5±0.6	5±0.6	4.5±0.2	5.1±0.3			
SM 17:0	13.9±1.4	12.1±1.3	6.2±0.4	6±0.3		**	
SM 18:0	515.6±37.8	434.7±47.5	419.3±40.6	458.5±24.1			
SM 18:1	39.5±2.3	32.7±3.7	34.8±2.9	36.7±1.6			
SM 19:0	10±2	9.5±1.1	2.5±0.7	2.6±0.9		**	
SM 21:0	0.2±0.2	0	1±0.4	1.5±0.9		*	
SM 21:1	0.1±0.1	0.8±0.5	0.2±0.1	0.3±0.1			
SM 23:0	26.4±3.1	19.8±2.5	12.9±1.5	15.3±0.8		**	
SM 23:1	12.9±1.8	7.2±1.3[#]	7.6±1.4	9±0.8	*		
SM 23:2	3.1±1.4	0.1±0.1[#]	0	0.5±0.4	*		
SM 24:0	66.3±5.3	54±4.1	41.1±3.8	46±3.1		**	
SM 24:1	118.8±10.3	104.4±9	71±8.4	86.2±5.5		**	
SM 24:3	13±8.2	9±4.9	0	0			
SM 26:0	5.1±0.6	3.5±0.5	2±0.7	3.3±0.6	*	*	
SM 26:1	3.7±0.5	2.2±0.7	3.2±0.5	3.7±0.3			
SM 26:2	0.3±0.3	2.1±0.9[#]	0	0.4±0.2			*

Sphingomyelin content in each group. Data is mean ± SEM, nmol/g. *$p<0.05$, **$p<0.01$ 2-way ANOVA followed by [#]$p<0.05$, [##]$p<0.01$ post hoc Sidak test was performed. N=6.

Table 7. Effect of diet and Acot7 overexpression on Phosphatidylglycerol species.

PG species	Control Chow	Acot7 Chow	Control HFD	Acot7 HFD	2-way ANOVA		
					Interaction	Diet	Acot7
PG (32:0) 16:0_16:0	2.2±0.1	2.6±0.2	3.6±0.3	3.9±0.4		**	
PG (34:0) 16:0_18:0	2.2±0.2	2±0.7	6.4±0.8	6.6±0.6		**	
PG (34:1) 16:0_18:1	76.7±2.8	65.4±3.6	81.5±3.2	81.5±5.9		*	
PG (34:2) 16:0_18:2	4.2±0.4	3.2±0.3	5.7±0.9	5.4±0.7		*	
PG (36:1) 18:0_18:1	7.5±0.8	7.2±0.7	12.7±0.8	12.9±1		**	
PG (36:2) 18:0_18:2	4.1±0.5	4.3±0.5	9.1±0.9	8.6±0.7		**	
PG (36:2) 18:1_18:1	4.9±0.4	3.5±0.4##	3.6±0.4	3.2±0.4			**
PG (36:3) 18:1_18:2	1.2±0.5	0.3±0.2#	0.9±0.2	1.6±0.2	**		

Phosphatidylglycerol content in each group. Data is mean ± SEM, nmol/g. *p<0.05, **p<0.01 2-way ANOVA followed by #p<0.05, ##p<0.01post hoc Sidak test was performed. N=6.

Table 8. Effect of diet and Acot7 overexpression on Phosphatidylserine species.

PS species	Control Chow	Acot7 Chow	Control HFD	Acot7 HFD	2-way ANOVA		
					Interaction	Diet	Acot7
PS (34:1) 16:0_18:1	2.5±0.5	0.9±0.6#	0	0.7±0.5	*	*	
PS (36:1) 18:0_18:1	36.8±2.3	32.3±2.2	27.8±1.1	24.9±1.3		**	
PS (36:2) 18:0_18:2	21.1±1.6	16.7±0.8	15.5±1.4	12.5±1		**	*
PS (36:2) 18:1_18:1	1.7±0.1	1.6±0.1	1.6±0.4	1.2±0.1			
PS (38:3) 18:0_20:3	16.5±1.2	13.4±0.5	11.4±0.6	11.8±0.6		**	
PS (38:4) 18:0_20:4	39.3±2	30.1±1.3#	46.7±2.3	45.4±2		**	
PS (38:5) 18:1_20:4	2.7±0.2	1.8±0.5	1.2±0.4	1.2±0.4		*	
PS (40:4) 18:0_22:4	18±1	13±0.6	33.3±1.8	31.3±2.1		**	
PS (40:5) 18:0_22:5	60.6±3	43.7±1.8	86±5.4	87.9±8.3		**	
PS (40:6) 18:0_22:6	236.1±8.4	179.1±9.8##	114±5.7	117.4±7.6	**	**	**

Phosphatidylserine content in each group. Data is mean ± SEM, nmol/g. *p<0.05, **p<0.01 2-way ANOVA followed by #p<0.05, ##p<0.01 post hoc Sidak test was performed. N=6.

Table 9. Effect of diet and Acot7 overexpression on Lysophosphatidylcholine species.

LPC species	Control Chow	Acot7 Chow	Control HFD	Acot7 HFD	2-way ANOVA		
					Interaction	Diet	Acot7
LPC 16:0	71.6±3.8	61.5±3.8	73.1±3.5	70.1±4			
LPC 18:0	39.6±2.2	32.5±1.8	47.1±2.5	45.6±3.6		**	
LPC 18:1	17.6±1	13.8±0.9#	13.6±1	11.6±0.6		**	*
LPC 18:2	27±2	21.3±1.1	18.3±2.2	14.4±1.4		**	*
LPC 20:4	17.8±1.1	15.1±0.7	32.8±3.3	32±1.9		**	
LPC 22:4	4.7±0.6	2.7±0.7##	4±0.1	4.8±0.4	**		
LPC 22:5	2.7±0.3	2.2±0.2	3±0.4	2.9±0.2			
LPC 22:6	11.4±0.8	9.9±0.9	4.6±0.5	4.8±0.5		**	

Lysophosphatidycholine content in each group. Data is mean ± SEM, nmol/g. * $p<0.05$, ** $p<0.01$ 2-way ANOVA followed by # $p<0.05$, ## $p<0.01$post hoc Sidak test was performed.

Table 10.Effect of diet and Acot7 overexpression on cardiolipin species.

CL species	Control Chow	Acot7 Chow	Control HFD	Acot7 HFD	2-way ANOVA		
					Interaction	Diet	Acot7
CL 72:5	66±16.4	72.4±8.5	111.8±12.4	66.9±10.1[#]	*		
CL 72:6	0.5±0.3	1.1±1.1	3.7±2.9	8.4±3.9			
CL 72:7	71.5±8.6	56.1±5.5	64±9.2	48.2±10.3			
CL 72:8	1446±86	970±72[#]	1163.7±159	1079.7±86.4			*
CL 74:10	8.4±2.2	7.8±0.9	14.6±3.2	12.8±3.2			
CL 74:7	141.9±18.2	115.4±9	111.9±19.8	67.9±8.2		*	*
CL 74:8	4.9±3.8	3±1.2	16.5±6.9	25.4±6.7		*	
CL 74:9	56±6.7	41.1±6.4	45.5±5.6	50.3±8.7			
CL 76:10	2.3±1.4	2.2±1.4	2.3±1.7	4.5±3.7			
CL 76:11	48.3±7.5	15.7±3.6[#]	26.1±5.9	39.1±9	**		
CL 76:12	7.4±5.2	24±2.6	11.7±2.7	16.4±5.2			

Cardiolipin content in each group. Data is mean ± SEM, nmol/g. *p<0.05, **p<0.01 2-way ANOVA followed by [#]p<0.05, [##]p<0.01post hoc Sidak test was performed. N=6.

Table 11. Effect of diet and Acot7 overexpression on Ceramide species.

Cer species	Control Chow	Acot7 Chow	Control HFD	Acot7 HFD	2-way ANOVA		
					Interaction	Diet	Acot7
Cer 16:0	3.1±0.3	4.2±0.3#	3±0.1	3.2±0.3			*
Cer 18:0	23.1±1	20.4±1.1	23.3±1.1	24.9±1.2		*	
Cer 19:0	0.5±0.1	0.5±0.1	0	0		**	
Cer 20:0	1.1±0.1	1.2±0	1±0.1	0.9±0.1			
Cer 22:0	1.9±0.2	1.6±0.1	1.9±0.1	1.4±0.1			*
Cer 22:2	0.6±0.1	0.5±0.1	0.4±0.1	0.3±0.1			
Cer 23:0	2.1±0.3	1.7±0.1	1.6±0.1	1.3±0.1			
Cer 24:0	7±1	6.5±0.6	6.8±0.6	5.8±0.5			
Cer 24:1	6.8±1	6.8±0.4	5.4±0.3	4.7±0.3		*	
Cer 24:2	0.8±0.1	0.9±0.1	0.8±0.1	0.8±0.1			
Cer 25:0	0.5±0.2	0.8±0.1	0.7±0.2	0.7±0.2			

Ceramide content in each group. Data is mean ± SEM, nmol/g. *p<0.05, **p<0.01 2-way ANOVA followed by #p<0.05, ##p<0.01 post hoc Sidak test was performed. N=6.

Table 12. Effect of diet and Acot7 overexpression on Cholesterol esters and free cholesterol.

Species	Control Chow	Acot7 Chow	Control HFD	Acot7 HFD	2-way ANOVA		
					Interaction	Diet	Acot7
CE 14:0	0	5.7±4.5	1.3±1	0			
CE 15:0	0	7.3±4.9	1.7±1.2	0			
CE 16:0	4.2±1.2	15±6.8	9.6±2.2	7.2±1			
CE 18:0	0	4±2.1	2.1±1.2	0	*		
CE 18:1	3.9±0.4	8.9±5	4±0.2	4.4±0.7			
CE 18:2	6.2±0.5	6.4±1.5	6.7±0.5	6.9±0.6			
CE 20:4	13.8±1	11.7±1	26±2.2	25±2.3		**	
CE 20:5	0.8±0.5	9.4±6.1	2.5±1.7	0.5±0.3			
CE 22:5	0	5±3.8	1.4±1.1	0			
Free Cholestrol	2282.5±179.9	1883.1±135.5	1972.5±114.8	1629.8±109.9		*	
Cholesterol esters and free cholesterol species content in each group. Data is mean ± SEM, nmol/g. * p<0.05, ** p<0.01 2-way ANOVA followed by $^{\#}$p<0.05, $^{\#\#}$p<0.01post hoc Sidak test was performed. N=6.							

Full immunoblots used for representative blots in Figure 1 of *"Increasing Acyl CoA thioesterase 7 activity alters phospholipid profile without effect on insulin action in skeletal muscle of rats."*

Figure 1

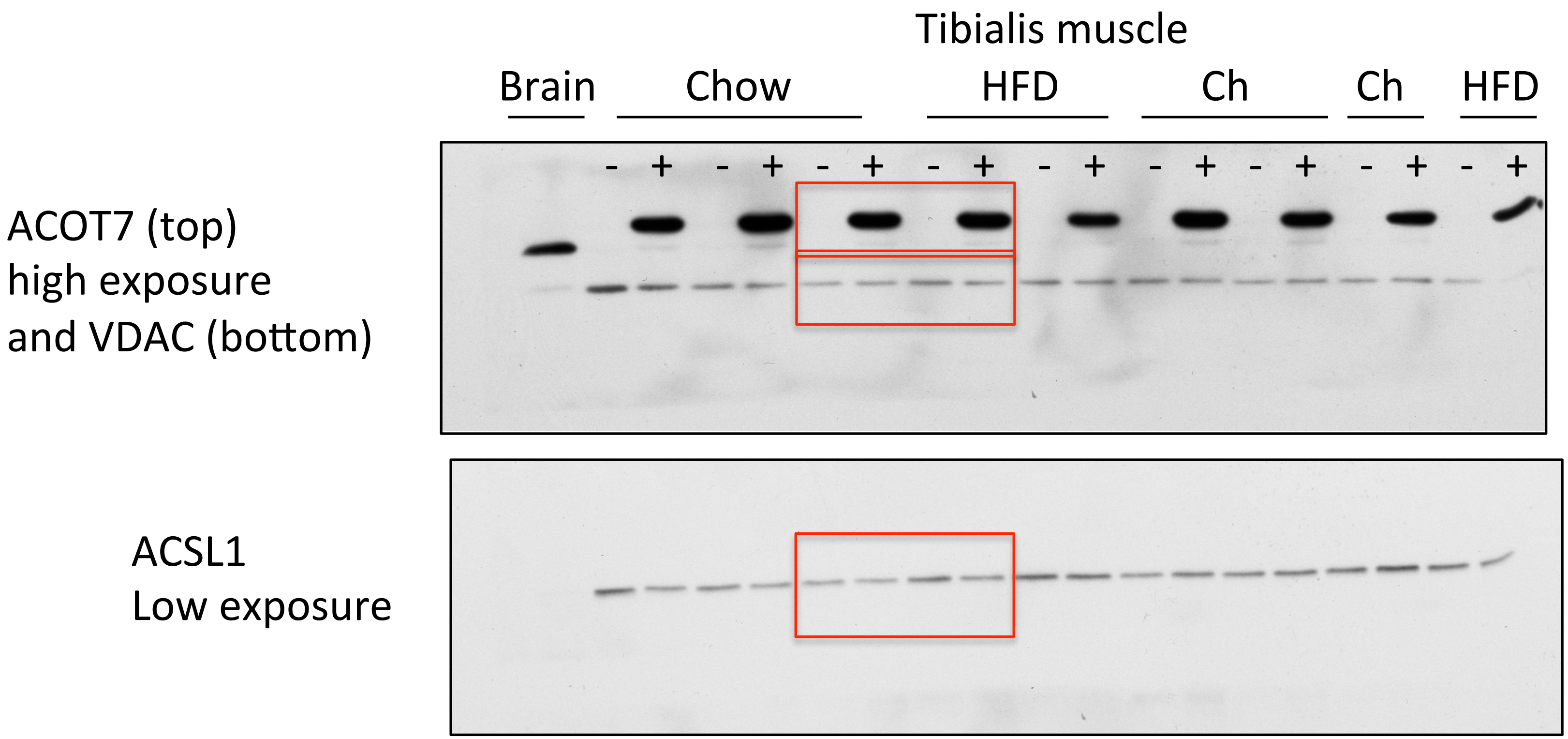

Full immunoblots used for representative blots in Figure 4 of *“Increasing Acyl CoA thioesterase 7 activity alters phospholipid profile without effect on insulin action in skeletal muscle of rats.”*

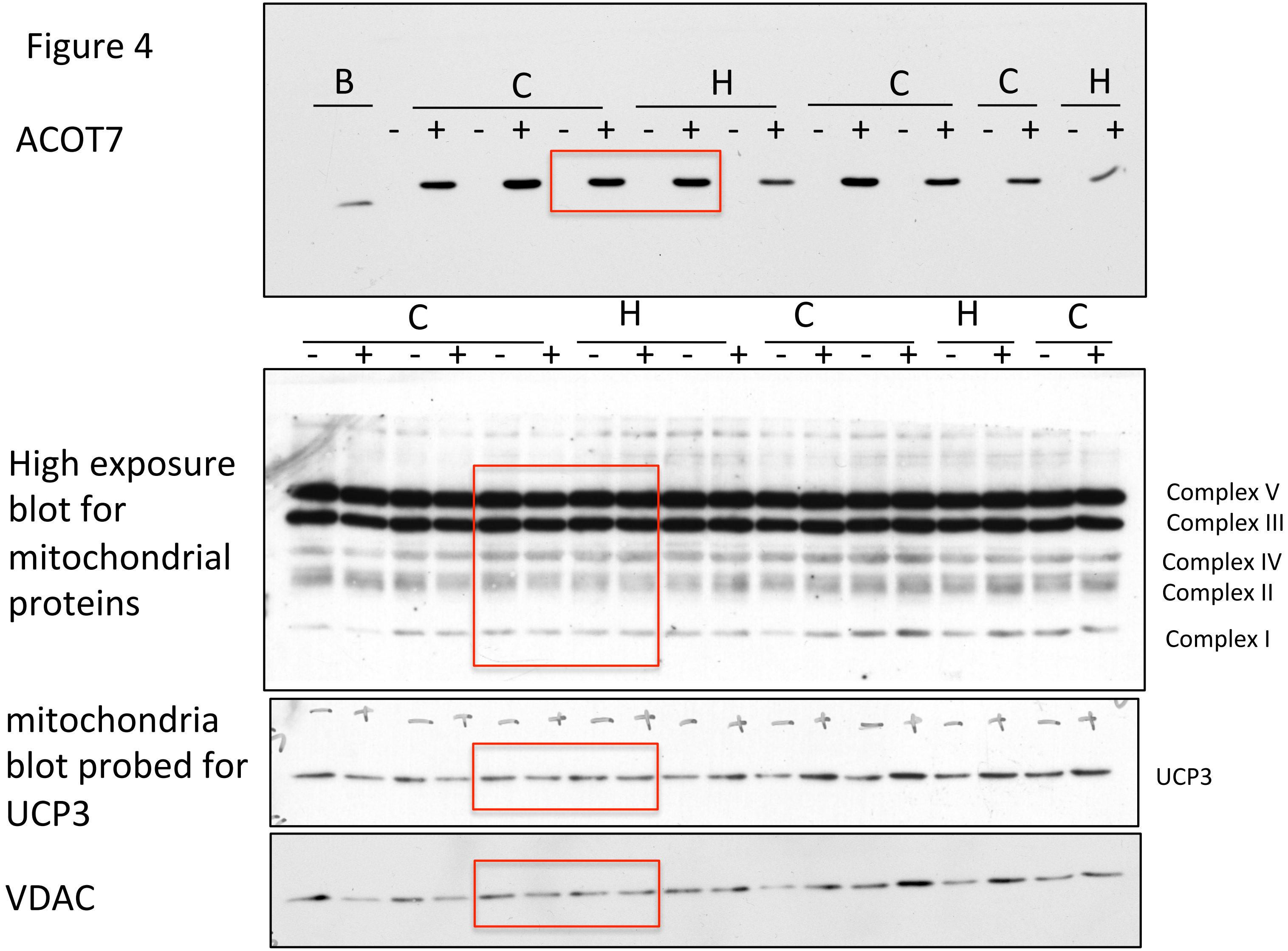

Supplement: Supplementary file 1 — Supplemental data [file 41598_2018_32354_MOESM1_ESM.pdf]
